# Complete Chloroplast Genomes of *Ranunculus arvensis* and *Ranunculus laetus*: Comparative Analysis and Phylogenetic Insights

**DOI:** 10.1002/ece3.73559

**Published:** 2026-04-29

**Authors:** Hui Li, Siyue Zhang, Abdullah, Sayed Afzal Shah, Yuhua Huang, Jingjing Jia, Liya Guo, Ying Cui, Jiahui Sun, Parviz Heidari, Xiaoxuan Tian

**Affiliations:** ^1^ State Key Laboratory of Chinese Medicine Modernization Tianjin University of Traditional Chinese Medicine Tianjin China; ^2^ Haihe Laboratory of Modern Chinese Medicine Tianjin China; ^3^ Department of Biological Sciences National University of Medical Sciences Rawalpindi Pakistan; ^4^ State Key Laboratory for Quality Ensurance and Sustainable Use of Dao‐di Herbs, National Resource Center for Chinese Materia Medica, China Academy of Chinese Medical Sciences Beijing P. R. China; ^5^ Faculty of Agriculture Shahrood University of Technology Shahrood Iran

**Keywords:** chloroplast genome, phylogeny, positive selection, Ranunculaceae, *Ranunculus*

## Abstract

*Ranunculus* L., the largest genus of Ranunculaceae, exhibits remarkable ecological diversity, yet genomic resources for this genus remain limited. Here we report the complete chloroplast (cp) genomes of *Ranunculus arvensis* L. and *Ranunculus laetus* Wall. ex Hook.f. & Thomson. The *R. laetus* assembly is the first cp genome reported for this species, whereas the *R. arvensis* assembly, generated from a Pakistani population, provides an independent accession that complements the recently released GenBank record PV621859 from China. Both genomes were analyzed comparatively against 22 previously published *Ranunculus* cp genomes. The cp genomes ranged from 154,474 to 158,314 bp in length and displayed a typical quadripartite structure. All analyzed species harbored 112 unique genes, comprising 78 protein‐coding genes, 30 transfer RNA genes, and four ribosomal RNA genes. The cp genomes were highly conserved in terms of gene order, guanine–cytosine content, codon usage, amino acid composition, and simple sequence repeats. Phylogeny‐aware selection analysis models (BUSTED, MEME, FUBAR) revealed that, despite overall purifying selection at the gene level, codon‐based FUBAR detected pervasive positive selection across ~34.6% of plastid genes; *matK* and *ycf1* emerged as the most prominent hotspots for both episodic and pervasive adaptation. Nucleotide diversity analyses revealed several highly variable regions, notably *rpl32–ndhF*, *ycf1*, and *ndhF*. Phylogenetic analyses based on complete cp genome sequences and complementary single‐gene trees resolved two major clades corresponding to biogeographic patterns: Clade I comprised predominantly Eurasian and East Asian taxa, whereas Clade II comprised a transcontinental assemblage of East Asian, North American, and Himalayan species. The *R. laetus* and *R. arvensis* were grouped in Clade II. Overall, these results elucidate the conserved genomic architecture, evolutionary dynamics, and phylogenetic relationships of *Ranunculus* cp genomes and provide valuable genomic resources for future studies on phylogeny, taxonomy, DNA barcoding, and population genetics within Ranunculaceae.

## Graphical abstract

Here, we de novo assembled the chloroplast genomes of *Ranunculus arvensis* and *Ranunculus laetus*, providing comprehensive comparative analysis with 21 congeners. The genomes exhibited conserved quadripartite structures with no major rearrangements, though lineage‐specific variations in *atpH* position were detected in two species. Evolutionary rate analysis detected pervasive positive selection in 27 plastid genes and episodic positive selection in six genes, with *matK* and *ycf1* showing the strongest signals.

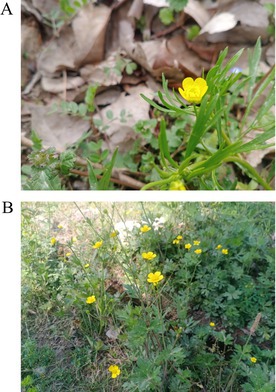

## 1 Introduction

The family Ranunculaceae comprises approximately 2500 species across 50 genera, thriving in temperate, subtropical, and high‐altitude tropical regions worldwide (POWO 2025; Wang et al. 2009). The family exhibits remarkable morphological and ecological diversity, making it an important model for research in taxonomy, evolutionary biology, and medicinal applications.

Within Ranunculaceae, *Ranunculus* L. is the most species‐rich genus, with 1760 accepted species distributed worldwide (POWO 2025). The genus occurs across a broad ecological range, from temperate to arctic and subantarctic zones, with notable diversity in major mountain systems, including the Himalayas, European Alps, and Mediterranean region (Emadzade et al. 2011; Paun et al. 2005).

*Ranunculus arvensis* L., commonly known as corn buttercup, is native to Europe, Western Asia, and North Africa, and has become naturalized in other regions, including North America and Australia (POWO 2025). Ethnobotanical studies report its traditional use in the treatment of rheumatism, arthritis, asthma, and skin disorders (Goo 2022; Shahzad Aslam et al. 2012). Its biological activity is attributed to flavonoids and other phenolic compounds with antioxidant and anti‐inflammatory properties, whereas protoanemonin accounts for its irritant and toxic properties (Al‐Snafi 2022).

*Ranunculus laetus* Wall. ex Hook.f. & Thomson is a perennial herb typically found in moist alpine meadows and wetlands. It is distributed across the Himalayan region, including Pakistan, India, Nepal, and the Tibet region of China (POWO 2025). In traditional medicine, this species has been used to treat intermittent fever, gout, and asthma (Shahzad Aslam et al. 2012). Phytochemical studies have identified flavonoids, tannins, saponins, glycosides, and steroids that underlie its antimicrobial and antioxidant activities (Goo 2022; Khalid et al. 2019). Although it is not currently classified as a threatened species, its sensitivity to environmental changes suggests potential vulnerability to habitat loss and climate change.

Although preliminary knowledge exists regarding the chemical profiles and traditional uses of *R. arvensis* and *R. laetus*, their evolutionary information at the genomic level remains scarce. The progress in high‐throughput sequencing has revolutionized our understanding of nuclear and organellar genomes (mitochondrial and chloroplast) while simultaneously contributing to the development of new therapeutic resources (Gao et al. 2023; Zhao et al. 2023). The chloroplast (cp) genome is particularly valuable for phylogenetic, evolutionary and DNA barcoding studies due to its uniparental (typically maternal) inheritance and general absence of recombination (Ahmed et al. 2020; Daniell et al. 2016; Zhang et al. 2023). Structurally, it features a quadripartite arrangement, consisting of a large single‐copy (LSC) region and a small single‐copy (SSC) region that are divided by a pair of inverted repeats (IRa and IRb) (Daniell et al. 2016). In most cases, cp genomes encode around 120 genes, including protein‐coding genes (CDS), transfer RNAs (tRNAs), and ribosomal RNAs (rRNAs) (Daniell et al. 2016).

Recent phylogenetic analyses have resolved evolutionary relationships and provided valuable data on cp genomes within Ranunculaceae (Paun et al. 2005). However, despite the approximately 1760 accepted species, complete chloroplast genomes have so far been reported for only about 22 species of *Ranunculus* have been reported, leaving the genus severely under‐sampled at the cp genome level. In the present study, we assembled and analyzed the complete chloroplast genomes of *R. arvensis* and *R. laetus* to: (1) characterize their genomic structure, (2) perform comparative analyses with congeners, (3) elucidate evolutionary dynamics within *Ranunculus*, and (4) reconstruct the phylogeny of the genus. Here, the present assembly of *R. laetus* constitutes the first complete chloroplast genome reported for this species. The *R. arvensis* cp genome described here represents an additional assembly from a Pakistani population that complements a recently released GenBank record (PV621859) from China, thereby providing the first opportunity to examine intraspecific cp genome variation across its geographic range.

## 2 Materials and Methods

### 2.1 Plant Collection, DNA Extraction, and Sequencing

**FIGURE 1:**
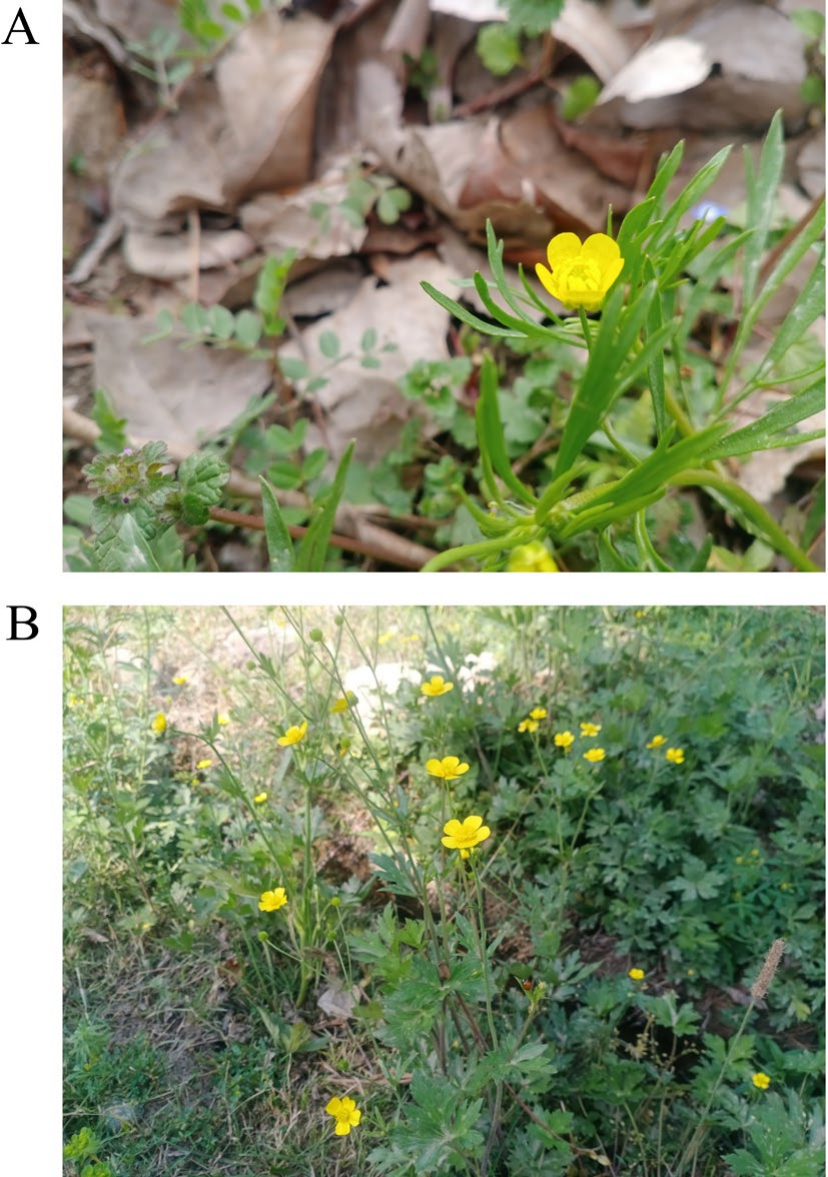
Representative photographs of species sequenced in the current study. (A) *Ranunculus arvensis* , an annual herb characterized by erect branched stems and small yellow flowers. (B) *Ranunculus laetus*, a perennial herb with slender stems bearing bright yellow flowers in its natural habitat.

Plant material of *Ranunculus arvensis* and *Ranunculus laetus* was collected from crop fields near the River Swat in Charbagh, Swat District, Khyber Pakhtunkhwa, Pakistan. The geographic coordinates of the collection sites were 34°49′48.0″ N, 72°25′48.0″ E for *R. arvensis* and 34°50′12.3″ N, 72°25′54.9″ E for *R. laetus* (Figure 1). No permission was required from the local authority or the central government of Pakistan for the collection of these plants or their parts, or for their use in research purposes. Species identification was confirmed by Dr. Sayed Afzal Shah. Representative voucher specimens were deposited in the herbarium of the National University of Medical Sciences under accession numbers NUMS00004 ( *R. arvensis* ) and NUMS00003 (*R. laetus*). Genomic DNA was extracted from silica‐dried leaf tissue using the Plant Genomic DNA Kit (TIANGEN BIOTECH, Beijing, China). Library preparation and sequencing were performed by Novogene (Tianjin, China) using the Illumina NovaSeq 6000 platform, generating 150 bp paired‐end reads. Quality filtering of raw reads was performed using fastp v0.23.1 (Chen et al. 2018) with the following parameters: ‐g ‐q 5 ‐u 50 ‐n 15 ‐l 150 ‐‐overlap_diff_limit 1 ‐‐overlap_diff_percent_limit 10. Paired reads were discarded if either read contained more than 10% ambiguous bases (N), more than 50% low‐quality bases (Q ≤ 5), or adapter contamination. Only high‐quality clean reads were retained for downstream analyses, with PHRED quality scores of Q20 and Q30 reaching 98.72% and 96.27% for *R. arvensis* , and 98.74% and 96.34% for *R. laetus*, respectively.

### 2.2 Chloroplast Genome Assembly and Annotation

The cp genomes were assembled *de novo* using GetOrganelle v1.7.5.3 (Jin et al. 2020) with default parameters for angiosperm chloroplast genomes. Initial annotation was performed using GeSeq v2.0.3 (Tillich et al. 2017) with the following settings: reference sequences from closely related *Ranunculus* species (*Ranunculus bungei* Steud., MK253468 and *Ranunculus chinensis* Bunge, NC_079824), BLAT search identity threshold of 85%, and HMM profile search for protein‐coding genes. Annotations were manually curated to verify start/stop codons, intron/exon boundaries, and gene boundaries using Geneious R8.1. All transfer RNA (tRNA) genes were validated using tRNAscan‐SE v2.0.7 (Chan and Lowe 2019) with default parameters for chloroplast genetic code. Ribosomal RNA (rRNA) genes were identified by BLAST comparison with reference genomes and confirmed by sequence similarity > 95%. The final annotated genomes were deposited in GenBank under accession numbers (PV364608 and PV364609). Circular genome maps were generated with Chloroplot (Zheng et al. 2020).

### 2.3 Chloroplast Genome Feature Analysis and Comparative Genomics

Comparative analyses were conducted between the assembled cp genomes and those of 21 additional *Ranunculus* species. Evaluations focused on genome size, gene composition, lengths of the large single‐copy (LSC), small single‐copy (SSC), and inverted repeat (IR) regions, as well as guanine–cytosine (GC) content. These comparisons were performed using Chloroplast Genome Analysis Suite (CGAS) v 1.0.1 (Abdullah et al. 2026) and further validated in Geneious R8.1 (Kearse et al. 2012). To illustrate intron‐containing genes, schematic diagrams of cis‐ and trans‐splicing genes were generated using CPGview (Liu et al. 2023). Furthermore, intron containing genes of all species were analyzed using CGAS v 1.0.1 to determine variations in their length among different species and then manually visualized in Geneious R8.1. Synteny and structural conservation were assessed with Mauve (Darling et al. 2004). The positions of IR boundaries were examined and visualized with CPJSdraw (Li et al. 2023).

### 2.4 Codon Usage, Amino Acid Frequency, and Microsatellites

Relative synonymous codon usage (RSCU) and amino acid frequencies were calculated using CGAS v 1.0.1 and further verified in Geneious R8.1 by looking at the codon usage and amino acid frequency of each species separately. Simple sequence repeats (SSRs) were detected with MISA‐web (https://pgrc.ipk‐gatersleben.de/misa/, accessed on March 15, 2026) (Beier et al. 2017) using the following thresholds: mononucleotide repeats ≥ 10, dinucleotide repeats ≥ 5, trinucleotide repeats ≥ 4, and tetra‐, penta‐, and hexanucleotide repeats ≥ 3. These results were also analyzed using CGAS v 1.0.1 and found to be similar to MISA results.

### 2.5 Substitution Patterns, Dynamics and Selective Pressure Analysis

To investigate substitution patterns, the LSC, SSC, and IR regions from all cp genomes were partitioned and aligned pairwise. *R. arvensis* was selected as the reference genome because it occupies a basal position. Substitution types were categorized, and the transition–transversion ratio was estimated using CGAS v 1.0.1, then manually verified by examining variations in Geneious R8.1. In addition, total number of substitutions were verified in DnaSP (Rozas et al. 2017).

To evaluate the evolutionary dynamics and selective pressures acting on protein‐coding genes across *Ranunculus* chloroplast genomes, selection analyses were conducted using HyPhy v2.5.7 (Kosakovsky Pond et al. 2020). Initially, codon‐based sequence alignments for all protein‐coding genes were generated utilizing MACSE v1 (Ranwez et al. 2018). Maximum‐likelihood phylogenetic trees were subsequently reconstructed for each aligned gene using FastTree (Price et al. 2010) under the GTR + G substitution model, serving as the required topological input for evolutionary modeling.

To capture distinct signatures of positive selection, we employed three complementary statistical frameworks: the branch‐site unrestricted statistical test for episodic diversification (BUSTED) to detect gene‐wide episodic selection (Murrell et al. 2015); the Mixed Effects Model of Evolution (MEME) to identify site‐specific episodic diversifying selection (Murrell et al. 2012); and the Fast Unconstrained Bayesian Approximation (FUBAR) to characterize sites under pervasive selection (Murrell et al. 2013). Notably, the entire workflow—ranging from GenBank file parsing and sequence extraction to the execution of selection algorithms and the aggregation of Excel‐formatted results with multiple‐testing corrections—was efficiently automated using CGAS v 1.0.1.

### 2.6 Identification of Polymorphic Loci and Phylogenetic Reconstruction

The nucleotide diversity of CDS, tRNA, rRNA, introns, and intergenic spacer regions were determined based on the analysis of all 23 chloroplast genomes included in this study using CGAS v 1.0.1. Furthermore, the nucleotide diversity results were independently validated and cross‐verified using DnaSP (Rozas et al. 2017). Comparative analysis demonstrated that the two analytical approaches yielded highly consistent results.

A maximum likelihood phylogenetic tree was reconstructed using the complete chloroplast genomes of 23 *Ranunculus* species, with *Oxygraphis glacialis* (NC_041538) designated as the outgroup. Sequence alignment was performed using MAFFT (Katoh and Standley 2013), producing a dataset of 84,671 nucleotide positions. Phylogenetic inference was conducted using IQ‐TREE v3 (Minh et al. 2020) under the best‐fit substitution model (K3Pu + F + R3), selected by ModelFinder (Kalyaanamoorthy et al. 2017). Among the aligned sites, 78,716 (92.97%) were conserved, and 3245 were parsimony‐informative. Node support was evaluated with 10,000 ultrafast bootstrap replicates and 10,000 SH‐aLRT tests, with the ‐bnni option enabled to refine tree topology. To further validate the phylogenetic framework and assess the resolving power of potential DNA barcodes, we extracted the 20 highly variable regions identified based on the nucleotide diversity analysis, comprising 10 CDS and 10 intergenic spacers (IGS). Individual maximum likelihood trees were constructed for each of the 20 regions using IQ‐TREE v3 under the same parameters. To synthesize the phylogenetic signal from the highly variable CDS, two additional strategies were employed: (1) a concatenated tree based on the combined alignment of the 10 CDS, and (2) a majority‐rule consensus tree computed directly from the 10 individual CDS trees. Specifically, the consensus tree was generated using the ‐con option in IQ‐TREE to summarize clade frequencies across the input trees, retaining branches that occurred in at least 50% of the individual gene phylogenies (Table S1). The resulting phylogeny was visualized using the Interactive Tree of Life (iTOL) platform (https://itol.embl.de/) (Letunic and Bork 2019).

## 3 Results and Discussion

### 3.1 Chloroplast Genome Organization and Comparative Genomics

**FIGURE 2:**
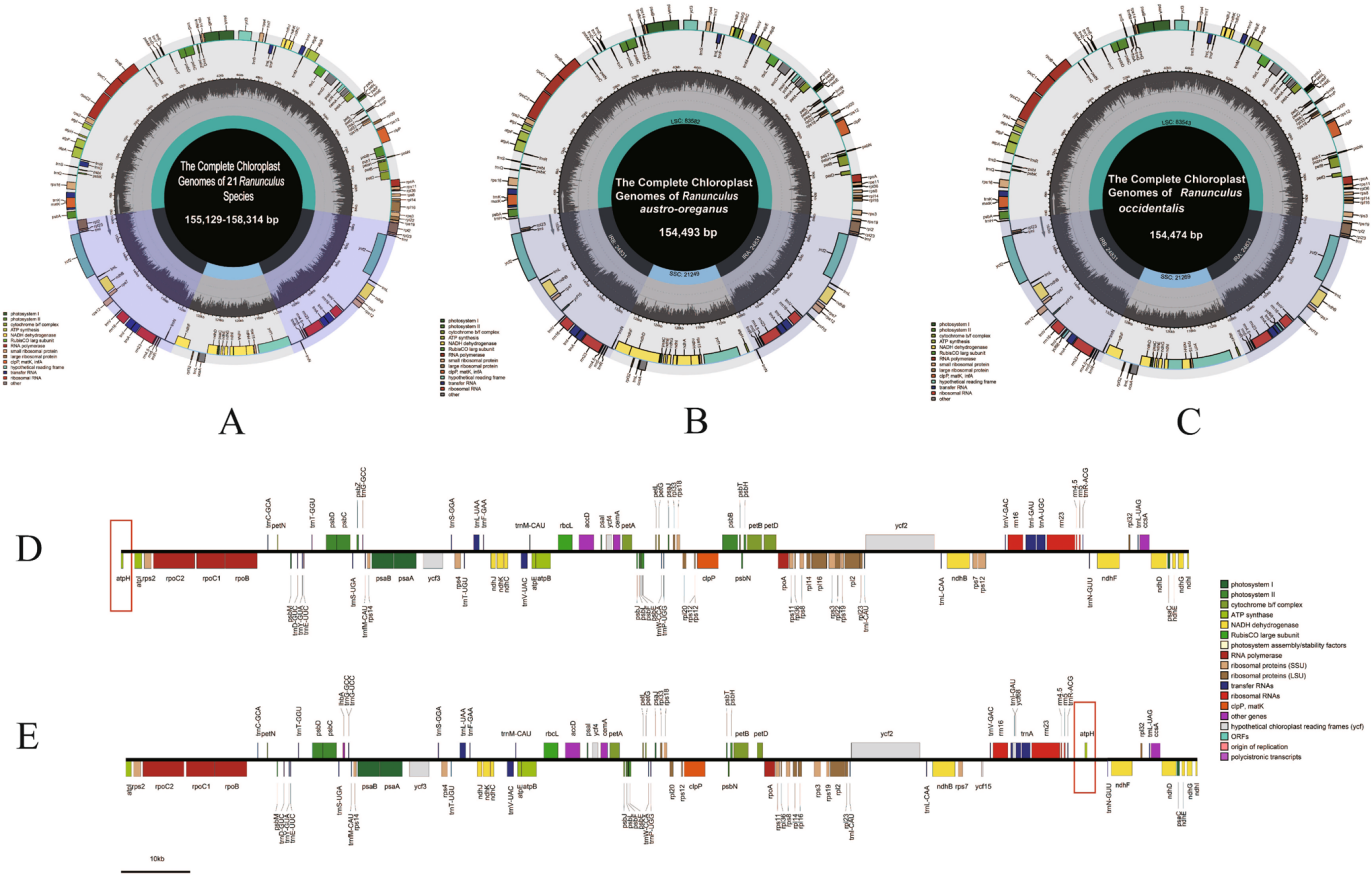
Circular chloroplast genome maps of *R. arvensis and R. laetus*. The circular genome maps illustrate the large single‐copy (LSC) region, the small single‐copy (SSC) region, and the pair of inverted repeats (IRa and IRb). Functional categories are distinguished by different colors as shown in the legend. Genes placed along the outer ring are oriented in the counterclockwise direction, while those located on the inner ring are oriented clockwise. The innermost gray histogram represents fluctuations in GC content throughout the genomes.

Whole‐genome sequencing generated 15.62 GB of data (22.45 million reads) for *R. arvensis* and 15.92 GB (22.90 million reads) for *R. laetus*. The chloroplast assemblies achieved high coverage, with average depths of 309.3× for *R. arvensis* and 292.8× for *R. laetus* (Figure S1; Figure S2). The chloroplast genomes of both *R. arvensis* and *R. laetus* exhibited the typical quadripartite structure, consisting of an LSC region, a SSC region, and two IRs (IRa and IRb). The complete cp genome was 156,925 bp in length for *R. arvensis* (LSC: 85,742 bp; SSC: 18,863 bp; IR: 26,160 bp) and slightly shorter, 156,633 bp, for *R. laetus* (LSC: 85,277 bp; SSC: 18,888 bp; IR: 26,234 bp). Comparative analysis with 21 additional *Ranunculus* genomes revealed highly conserved gene content, intron composition, and structural organization. However, complete cp genome length varied from 154,474 bp ( *Ranunculus occidentalis* ) to 158,314 bp ( *Ranunculus trichophyllus* ) across the genus (Table 1; Figure 2).

**TABLE 1:** Comparison of the chloroplast genomes of 23 *Ranunculus* species.

Species	Genome length (bp)				GC content (%)				GC content of genes (%)			Accession no.
	Total	LSC	SSC	IR	Total	LSC	SSC	IR	tRNA	rRNA	CDS	
***Ranunculus arvensis***	156,925	85,742	18,863	26,160	37.71	35.84	31.02	43.18	53.03	55.4	38.19	PV364608
*Ranunculus austro‐oreganus*	154,493	83,582	21,249	24,831	37.79	35.9	31.58	43.63	53.02	55.43	37.9	KX639503
*Ranunculus bungei*	156,082	85,436	19,942	25,352	37.83	36.02	31.25	43.46	52.89	55.43	38.19	MK253468
*Ranunculus cantoniensis*	155,154	84,634	21,665	24,427	37.91	36.03	31.98	43.8	53.11	55.45	38.22	NC045920
*Ranunculus cassubicifolius*	156,233	85,629	19,826	25,389	37.93	36.15	31.39	43.46	52.81	55.47	38.29	NC077490
*Ranunculus chinensis*	155,289	84,814	18,887	25,794	37.92	36.03	31.17	43.49	53.11	55.45	38.23	NC079824
*Ranunculus japonicus*	156,981	85,454	18,897	26,315	37.72	35.96	31.33	42.88	52.88	55.45	38.21	MZ169045
*Ranunculus kadzusensis*	158,301	84,973	17,638	27,845	37.83	36.11	31.25	42.55	52.89	55.43	38.27	PQ246022
***Ranunculus laetus***	156,633	85,277	18,888	26,234	37.77	35.98	31.36	43.01	52.88	55.45	38.21	PV364609
*Ranunculus macranthus*	155,129	84,638	18,909	25,791	37.88	36	31.01	43.49	53.11	55.45	38.18	NC008796
*Ranunculus membranaceus*	156,028	85,491	19,815	25,361	37.91	36.1	31.45	43.48	52.77	55.45	38.3	NC065303
*Ranunculus mongolicus*	158,309	84,974	17,637	27,849	37.83	36.11	31.23	42.54	52.89	55.43	38.27	OR625576
*Ranunculus monophyllus*	155,973	85,345	19,790	25,419	37.91	36.16	31.37	43.39	52.81	55.45	38.28	OR625578
*Ranunculus occidentalis*	154,474	83,543	21,269	24,831	37.8	35.9	31.62	43.63	53.02	55.45	38.12	NC031651
*Ranunculus pekinensis*	156,139	85,431	19,974	25,367	37.83	36.02	31.25	43.45	52.89	55.43	38.19	NC060613
*Ranunculus polyrhizos*	156,000	85,414	19,716	25,435	37.88	36.08	31.42	43.4	52.81	55.45	38.25	OR625579
*Ranunculus sardous*	155,243	85,252	18,913	25,539	37.86	36.01	31.02	43.49	53.11	55.45	38.17	OZ222637
*Ranunculus sceleratus*	156,329	85,840	19,885	25,302	37.93	36.07	31.7	43.55	52.89	55.43	38.33	NC080350
*Ranunculus silerifolius var. silerifolius*	155,368	84,882	18,880	25,803	37.9	36.02	31.12	43.49	53.11	55.45	38.23	ON462450
*Ranunculus tanguticus*	156,186	85,627	19,785	25,387	37.9	36.07	31.5	43.48	52.83	55.45	38.28	OR625580
*Ranunculus ternatus*	156,003	85,397	19,856	25,375	37.86	36.06	31.3	43.46	52.85	55.45	38.25	NC081908
*Ranunculus trichophyllus*	158,314	84,945	17,635	27,867	37.84	36.12	31.18	42.56	52.89	55.43	38.27	OR625577
*Ranunculus yunnanensis*	156,050	85,556	19,772	25,361	37.92	36.09	31.53	43.49	52.9	55.45	38.29	NC063514

*Note:* The bold font indicates the species that were sequenced in the current study.

Abbreviations: IR, inverted repeat; LSC, large single copy; SSC, small single copy.

**FIGURE 3:**
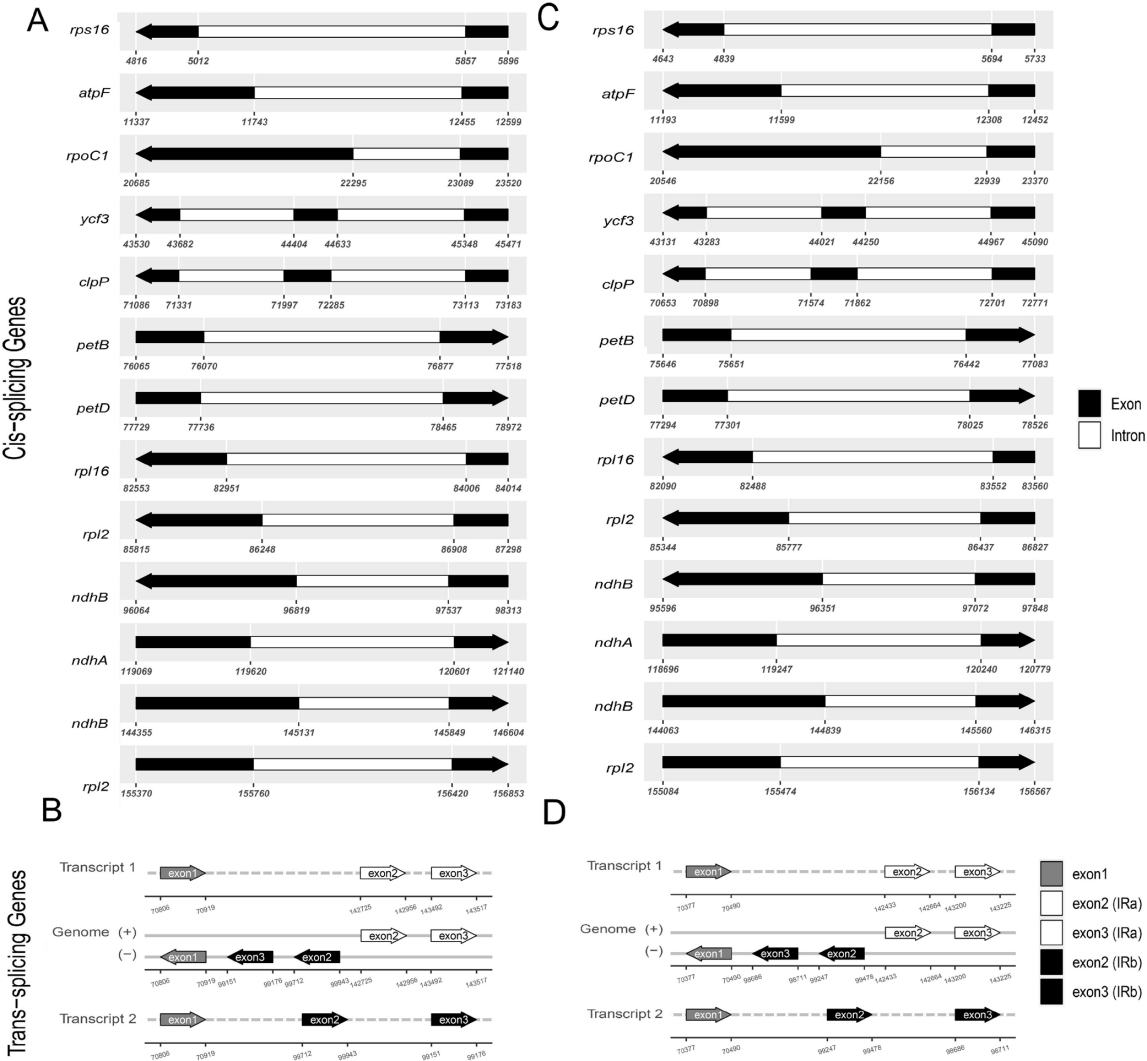
Schematic map of the cis‐splicing and trans‐splicing genes in the chloroplast genomes of *R. arvensis and R. laetus*. The cis‐splicing genes of *R. arvensis* . The trans‐splicing genes of *R. arvensis* . The cis‐splicing genes of *R. laetus*. The trans‐splicing genes of *R. laetus*.

All genomes contained 112 unique genes: 78 protein‐coding genes, 30 tRNAs, and 4 rRNAs. Sixteen genes were duplicated in the IR regions, including five protein‐coding genes (*rpl2*, *ycf2*, *rpl23*, *ndhB*, *rps7*), seven tRNA genes (*trnA‐UGC*, *trnI‐CAU*, *trnI‐GAU*, *trnL‐CAA*, *trnN‐GUU*, *trnR‐ACG*, *trnV‐GAC*), and four rRNA genes (*rrn16*, *rrn23*, *rrn4.5*, *rrn5*). Among these duplicated genes, the protein‐coding genes *rpl2* and *ndhB*, along with the tRNA genes *trnA‐UGC* and *trnI‐GAU*, contained introns. In total, 17 genes harbored introns, comprising 12 protein‐coding (*atpF*, *petB*, *rps16*, *rps12*, *rpl2*, *ndhA*, *rpl16*, *ndhB*, *rpoC1*, *ycf3*, *petD*, *clpP*) and 5 tRNA genes (*trnV‐UAC*, *trnK‐UUU*, *trnI‐GAU*, *trnL‐UAA*, *trnA‐UGC*). Among the CDS genes, 11 underwent cis‐splicing, including nine with a single intron and two (*ycf3* and *clpP*) with two introns each (Figure 3). Additionally, the *rps12* gene also contains two introns; however, it is trans‐spliced, with exon 1 located in the LSC region and exons 2 and 3 located in the IR regions (Table 2, Figure 3).

**TABLE 2:** Functional classification of genes in the chloroplast genome of *Ranunculus* species.

Genes	Group of genes	Name of genes	Amount
**Self‐replication**	Large subunit of ribosome	*rpl14*, *rpl16* *, rpl2*^,^ ^a^, *rpl20*, *rpl22*, *rpl23* ^a^, *rpl32*, *rpl33*, *rpl36*	11
	Small subunit of ribosome	*rps11*, *rps12* *, *rps14*, *rps15*, *rps16* *, *rps18*, *rps19*, *rps2*, *rps3*, *rps4*, *rps7* ^a^, *rps8*	13
	DNA dependent RNA polymerase	*rpoA*, *rpoB*, *rpoC1* *, *rpoC2*	4
	rRNA genes	*rrn16* ^a^, *rrn23* ^a^, *rrn4.5* ^a^, *rrn5* ^a^	8
	tRNA genes	trnA‐UGC*^,^ ^a^, *trnC‐GCA*, *trnD‐GUC*, *trnE‐UUC*, *trnF‐GAA*, *trnG‐GCC*, *trnG‐UCC*, *trnH‐GUG*, *trnI‐CAU* ^a^, trnI‐GAU*^,^ ^a^, *trnK‐UUU* *, *trnL‐CAA* ^a^, *trnL‐UAA* *, *trnL‐UAG*, *trnM‐CAU*, *trnN‐GUU* ^a^, *trnP‐UGG*, *trnQ‐UUG*, *trnR‐ACG* ^a^, *trnR‐UCU*, *trnS‐GCU*, *trnS‐GGA*, *trnS‐UGA*, *trnT‐GGU*, *trnT‐UGU*, *trnV‐GAC* ^a^, *trnV‐UAC* *, *trnW‐CCA*, *trnY‐GUA*, *trnfM‐CAU*	37
**Photosynthesis**	Photosystem I	*psaA*, *psaB*, *psaC*, *psaI*, *psaJ*	5
	Photosystem II	*psbA*, *psbB*, *psbC*, *psbD*, *psbE*, *psbF*, *psbH*, *psbI*, *psbJ*, *psbK*, *psbL*, *psbM*, *psbN*, *psbT*, *psbZ*	15
	NADPH dehydrogenase	*ndhA* *, ndhB*^,^ ^a^, *ndhC*, *ndhD*, *ndhE*, *ndhF*, *ndhG*, *ndhH*, *ndhI*, *ndhJ*, *ndhK*	12
	Cytochrome b/f complex	*petA*, *petB* *, *petD* *, *petG*, *petL*, *petN*	6
	Subunits of ATP synthase	*atpA*, *atpB*, *atpE*, *atpF* *, *atpH*, *atpI*	6
	Large subunit of Rubisco	*rbcL*	1
	Photosystem I assembly proteins	*ycf3* *, *ycf4*	2
**Other genes**	Protease	*clpP* *	1
	Maturase	*matK*	1
	Envelope membrane protein	*cemA*	1
	Subunit of Acetyl‐CoA‐carboxylase	*accD*	1
	C‐type cytochrome synthesis gene	*ccsA*	1
	Conserved open reading frames	*ycf1*, *ycf2* ^a^	3
Total number of genes			128

* Indicates genes containing introns.

^a^ Next to specific genes denote those duplicated in the IR regions.

The intron analysis showed high conservation among *Ranunculus* species (Table S2). Analysis of the 23 chloroplast genomes revealed highly consistent GC content across all structural regions as the overall GC content was 37.71%–37.93%, IR was 42.54%–43.80%, LSC was 35.84%–36.16%, and SSC was 31.01%–31.98% (Table 1).

The chloroplast genomes of *Ranunculus* species demonstrated remarkable evolutionary conservation, with all 23 genomes conforming to the ancestral Type I structure described by Zhai et al. (2019) for Ranunculaceae. This structural uniformity indicates exceptional stability since divergence from their common ancestor, suggesting minimal large‐scale rearrangements have occurred during *Ranunculus* diversification. The observed size variation (154,474–158,314 bp) primarily reflects minor length differences in intergenic spacer regions rather than major structural reorganizations.

The exceptionally high conservation of gene content, intron composition, and GC distribution patterns across *Ranunculus* species aligns with broader patterns observed in other Ranunculaceae members and angiosperm lineages (Chen et al. 2025; Zhai et al. 2019). The elevated GC content in IR regions, attributed to the presence of rRNA genes, follows typical angiosperm chloroplast genome architecture and has been consistently reported across diverse plant families (Li, Jia, et al. 2025; Zhai et al. 2019).

The pseudogenization of *infA* mirrors broader evolutionary trends observed not only in Ranunculaceae but also in other angiosperm lineages such as Malvaceae, reflecting ongoing functional gene transfer from chloroplast to nuclear genomes (Abdullah et al. 2020; Li, Jia, et al. 2025; Zhai et al. 2019). This pattern of gene loss represents a common evolutionary trajectory in angiosperm chloroplast genome evolution.

### 3.2 Analysis of Collinearity

**FIGURE 4:**
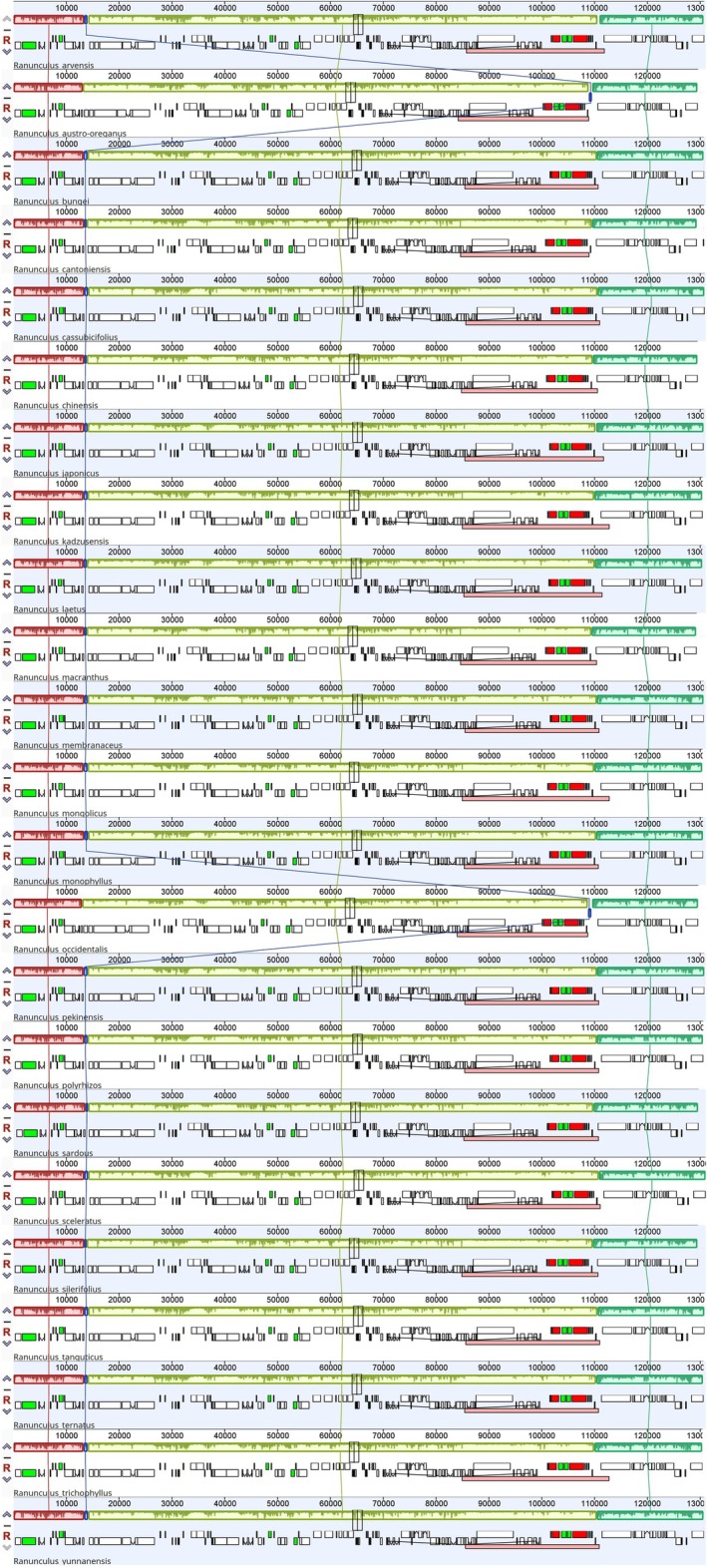
Mauve alignment in the chloroplast genomes of 23 *Ranunculus* species. The collinear block represents similarities among the species in gene content and arrangement.

Collinearity analysis of chloroplast genomes from 23 *Ranunculus* species revealed exceptionally high synteny across the genus. However, a notable exception was observed in *R. occidentalis* and *Ranunculus austrooreganus* , where the *atpH* gene relocated from its typical position at ~15 kb in the LSC region to ~120 kb in the SSC region (Figure 4). This rearrangement was previously reported in a comparative analysis of 11 *Ranunculus* species (Kim et al. 2023), and despite our expanded dataset of 23 species, this structural variation remained unique to these two species, confirming its lineage‐specific nature.

The *atpH* translocation represents a rare but significant departure from the otherwise highly conserved gene organization within *Ranunculus*. While the possibility of assembly artifacts cannot be entirely dismissed and warrants further validation through independent sequencing approaches, the consistency of this finding across studies suggests it represents a genuine evolutionary event. Such lineage‐specific rearrangements are not unprecedented in chloroplast genomes; similar patterns have been documented in other genera, including *Triumfetta*, where six genes were inverted in a single species, and *Daphne*, which exhibited two large inversions—one species‐specific and another serving as a synapomorphic character with phylogenetic utility (Abdullah et al. 2026; Yan et al. 2025).

This structural variation in *R. occidentalis* and *R. austrooreganus* may provide valuable phylogenetic markers for understanding evolutionary relationships within the *R. occidentalis* complex and could serve as synapomorphic characters for these lineages. The rarity of such rearrangements in otherwise highly conserved chloroplast genomes makes them particularly informative for phylogenetic reconstruction and evolutionary studies within *Ranunculus*.

### 3.3 Inverted Repeat Contraction and Expansion

**FIGURE 5:**
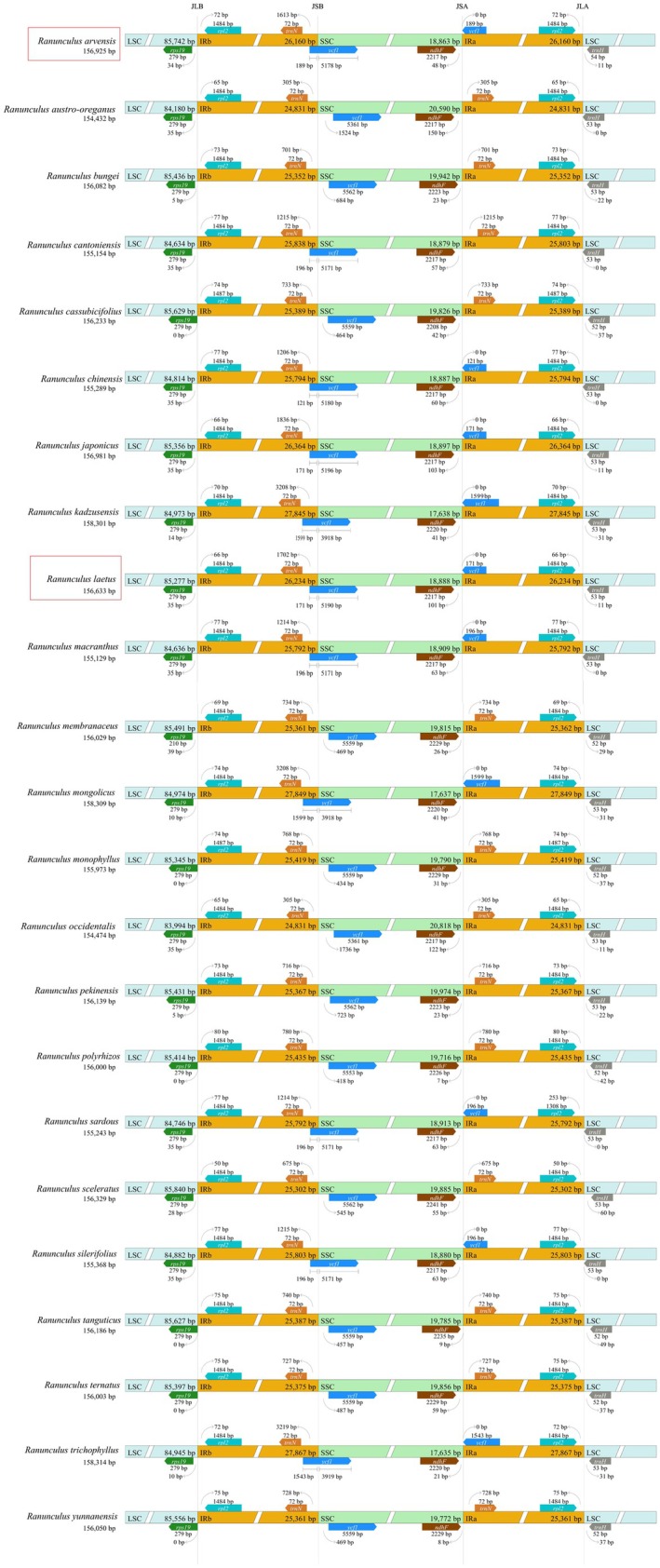
Comparison of inverted repeat expansion and contraction of 23 *Ranunculus* species. JLB = LSC/IRb, JSB = IRb/SSC, JSA = SSC/IRa, and JLA = IRa/LSC.

IR boundary analysis of chloroplast genomes across 23 *Ranunculus* species revealed a highly conserved quadripartite structure with minor species‐specific variations, consistent with previous studies (Kim et al. 2023) (Figure 5). Both IRb and IRa regions contained an intact *rpl2* gene, with distances ranging from 50 bp ( *Ranunculus sceleratus* ) to 80 bp (*Ranunculus polyrhizos*) from the LSC/IRb (JLB) and LSC/IRa (JLA) junctions. The *ndhF* gene was entirely located within the SSC region, with distances from the IRa/SSC junction (JSA) ranging from 7 bp in *R. polyrhizos* to 150 bp in *R. austrooreganus* .

At the IRb/SSC junction (JSB), the *ycf1* gene exhibited two distinct organizational patterns. In one group (e.g., *R. arvensis* , *Ranunculus cantoniensis*, *R. chinensis* , *Ranunculus japonicus*; 11 species total), *ycf1* spanned the boundary, generating a pseudogene (*ycf1Ψ*) in the IRa region ranging from 121 bp ( *R. chinensis* ) to 1599 bp (*Ranunculus kadzusensis* and *Ranunculus mongolicus*). In the second group (e.g., *R. austrooreganus* , *R. bungei*, *Ranunculus cassubicifolius*, *Ranunculus membranaceus*; 12 species total), *ycf1* was positioned entirely within the SSC region, with no corresponding pseudogene in IRa. The *rps19* gene was consistently located in the LSC region, with distances from JLB ranging from 0 bp to 35 bp across all species.

IR expansion and contraction events have been previously reported to be associated with gene duplication, deletion, or pseudogenization (Abdullah, Li, et al. 2026, 2020; Henriquez et al. 2020; Li et al. 2023; Zhai et al. 2019). In this study, *Ranunculus* species showed high conservation, and the observed IR boundary variations did not lead to gene duplications, with only the *ycf1* pseudogene formation at junction JSA representing a notable structural consequence. The IR contraction and expansion patterns were not clade‐specific on the phylogenetic tree in this study. Similarly, analyses of other Ranunculaceae genera did not reveal clade‐specific IR patterns (Zhai et al. 2019). However, in other taxa such as *Medicago*, *Berberis*, and the subfamily Asclepiadoideae of Apocynaceae, IR expansion and contraction have been linked to population genetics and phylogenetic differentiation (Choi et al. 2020; Li, Abdullah, et al. 2025). Future studies of *Ranunculus* incorporating broader taxonomic sampling may provide additional insights, as among the approximately 1760 described species, only 23 were analyzed in this study.

### 3.4 Codon Usage, Amino Acid Frequency, and Simple Sequence Repeat Analysis

**FIGURE 6:**
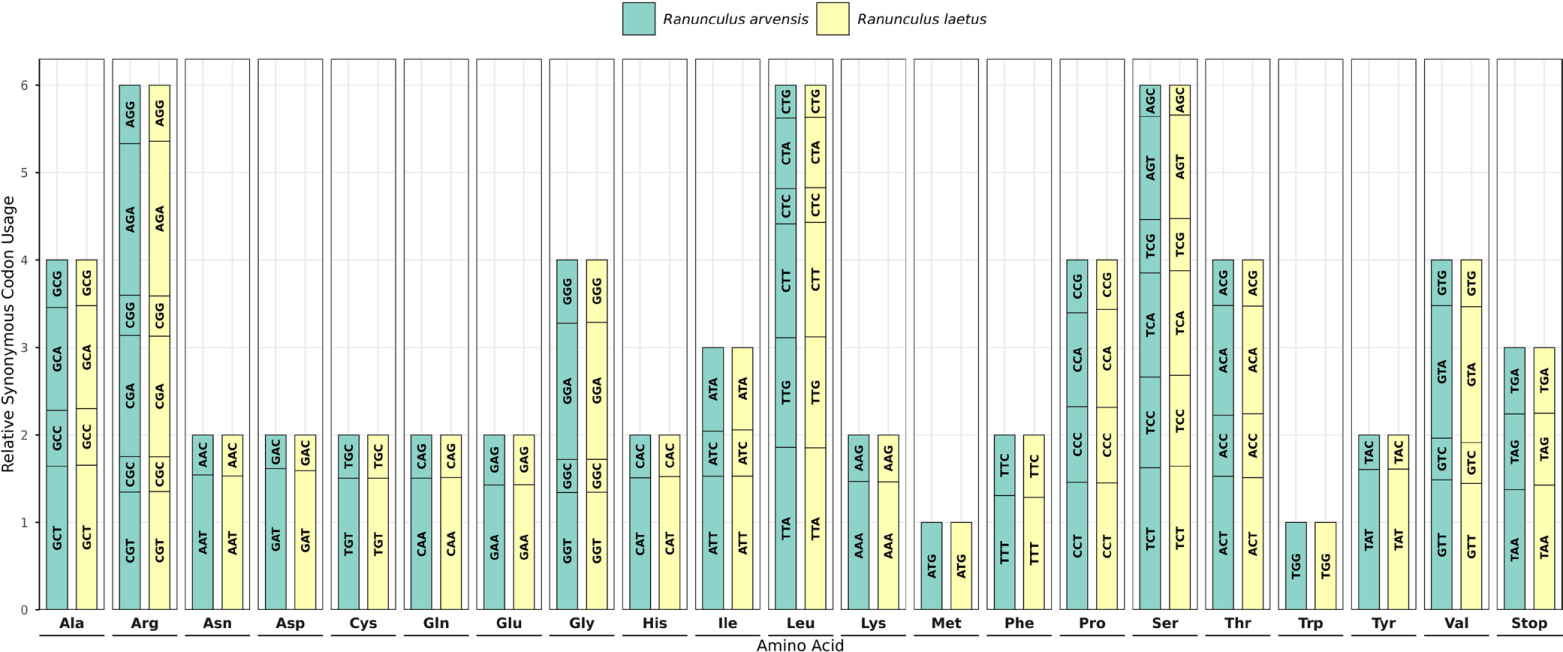
Codon usage bias analysis in the chloroplast genomes of *R. arvensis and R. laetus*. The x‐axis lists the amino acids, and the y‐axis shows the relative synonymous codon usage (RSCU) values. Each codon is labeled within its corresponding bar.

Analysis of RSCU revealed strong preference for codons ending in A or T across most amino acids. As illustrated in Figure 6 and Table S3, synonymous codons terminating in A or T consistently displayed RSCU values exceeding 1.0, indicating bias. In contrast, those ending in C or G were typically underrepresented. This bias reflects the elevated A/T content of coding regions, consistent with patterns reported in *Ranunculus* and other plant lineages (Ji et al. 2023; Raubeson et al. 2007). Regarding amino acid frequencies, leucine and isoleucine were the most abundant, whereas cysteine was the least common (Table S4). This distribution mirrors trends in other angiosperms, including Ranunculaceae species (Abdullah et al. 2020; Henriquez et al. 2020).

SSR analysis across 23 *Ranunculus* species revealed between 43 and 61 simple sequence repeats. Mononucleotide repeats predominated (23–40), followed by dinucleotide (5–13) and tetranucleotide repeats (4–10) (Table S5 and Table S6). Trinucleotide repeats ranged from 1 to 9, while pentanucleotide (0–3) and hexanucleotide repeats (0–1) were rare. Most repeats were A/T‐rich, consistent with the AT‐bias characteristic of chloroplast genomes in other Ranunculaceae species (Ji et al. 2023; Raubeson et al. 2007; Xia et al. 2022).

### 3.5 Substitution Analysis

Analysis of nucleotide substitutions in the chloroplast genomes revealed the most common types: A → G, G → A, C → T, and T → C. These patterns varied among *Ranunculus* species and across genomic regions. Substitution counts were highest in the LSC region (1669–3237), followed by the SSC (648–1305) and IR regions (66–681). The transition‐to‐transversion (Ts/Tv) ratios also varied: the IR region displayed the highest values (1.01–1.75), followed by the SSC (1.11–1.39) and LSC (0.99–1.30). Detailed results are provided in Table S7.

The observed predominance of transitions (Ts) over transversions (Tv) in the *Ranunculus* chloroplast genomes, resulting in Ts/Tv ratios greater than 1, aligns with previous reports (Cao et al. 2018; Rehman et al. 2021). However, Ts/Tv ratios below 1 have also been reported in some plant lineages (Abdullah et al. 2019 and citations therein).

### 3.6 Evolutionary Dynamics and Selective Pressure Analysis of Protein‐Coding Genes in *Ranunculus*

To comprehensively and robustly evaluate the evolutionary dynamics of *Ranunculus* chloroplast genes, we employed the phylogeny‐aware HyPhy software suite to conduct a multi‐model selection pressure analysis integrating both gene‐level and codon‐site‐level assessments.

Gene‐wide selection analysis using BUSTED revealed that although the vast majority of chloroplast genes are constrained by strong pervasive purifying selection (indicated by the initial global dN/dS estimations, e.g., *psaA* ω = 0.0141, *psaB* ω = 0.0352), six genes (*matK, petD, rbcL, rpl20, rpoA*, and *ycf1*) successfully exhibited statistically significant signals of episodic positive selection across the phylogeny. At the codon level, MEME initially identified up to 51 individual sites under episodic diversifying selection ($*p* < 0.1$). After applying a rigorous global false discovery rate (FDR) correction, four codon sites located in *ccsA*, *matK*, *ndhF*, and *ycf1* remained statistically significant (FDR < 0.05). Furthermore, FUBAR analysis, which detects pervasive selection, identified positively selected codons (posterior probability PP > 0.9) distributed across 27 genes (accounting for ~34.6% of the annotated plastid genes): *accD*, *atpB*, *atpF*, *atpI*, *ccsA*, *cemA*, *matK*, *ndhA*, *ndhB*, *ndhD*, *ndhF*, *ndhK*, *psaI*, *psbB*, *rbcL*, *rpl20*, *rpl22*, *rpoA*, *rpoB*, *rpoC1*, *rpoC2*, *rps18*, *rps3*, *rps4*, *ycf1*, *ycf2*, and *ycf4* (Tables S8 and S9). Notably, some genes identified as positively selected by BUSTED (e.g., *rbcL*, *rpl20* and *rpoA*) did not yield significant individual sites in MEME after FDR correction, whereas FUBAR successfully captured additional low‐signal pervasive codons within them. These results highlight the complementary nature of these methods, providing a highly comprehensive landscape of selective pressures in *Ranunculus*.

Specifically, BUSTED and MEME are highly sensitive to “episodic” selection—brief periods of rapid diversification restricted to specific branches or individual codons. In our study, several genes, notably *rbcL* (encoding the large subunit of Rubisco), *rpl20*, *rpoA*, and *petD*, showed robust gene‐wide evidence of episodic selection in BUSTED. The episodic adaptation of *rbcL*, in particular, has been frequently documented across diverse plant lineages as a critical evolutionary response to fluctuating temperatures and atmospheric CO2 concentrations (Galmés et al. 2014). The fact that these genes lacked significant codon‐level support in MEME after stringent FDR correction underscores the differences in statistical power: episodic selection often drives adaptations across a subset of sites that may not individually pass highly conservative significance thresholds, yet collectively contribute to functional divergence (Murrell et al. 2015).

Most strikingly, our FUBAR analysis revealed that 27 out of the 78 analyzed plastid genes (~34.6%) harbor sites evolving under pervasive positive selection. This relatively high proportion strongly aligns with recent comprehensive cp genome‐wide studies suggesting that adaptive evolution in chloroplasts is far more widespread than traditionally assumed. For example, a major study on PACMAD grasses by Piot et al. (2018), discovered that precisely one‐third of the plastid genes evolved under positive selection. Given that the genus *Ranunculus* has successfully colonized a highly heterogeneous array of habitats—ranging from aquatic ecosystems to terrestrial and high‐alpine zones (Emadzade et al. 2011)—this pervasive selective signature across roughly one‐third of the cp genome likely reflects continuous genomic tuning to these diverse and extreme environmental pressures. The remarkable consistency between our findings in *Ranunculus* and the evolutionary patterns observed in PACMAD grasses reinforces the emerging consensus that cp genomes serve as crucial reservoirs of adaptive variation.

Among these adaptive targets, the repeated identification of *matK* and *ycf1* across multiple independent methods (BUSTED, MEME, and FUBAR) is particularly notable. Both genes are widely recognized as rapidly evolving regions and prominent hotspots of adaptive evolution in angiosperms (Dong et al. 2015; Hilu and Liang 1997). Biologically, *matK* encodes a splicing maturase essential for the post‐transcriptional processing of chloroplast group II introns, while *ycf1* (Tic214) has been identified as an indispensable component of the chloroplast protein import translocon (TIC complex) (Nakai 2015; Zoschke et al. 2010). The robust positive selection detected in these genes suggests that modifications in RNA splicing efficiency and protein import dynamics may have been critical adaptive strategies. Overall, the integration of multiple analytical frameworks not only validates the robustness of our selection inferences but also highlights that adaptive evolution within key plastid genes has likely played an integral role in the evolutionary success and ecological diversification of *Ranunculus*.

### 3.7 Nucleotide Diversity Analysis

**FIGURE 7:**
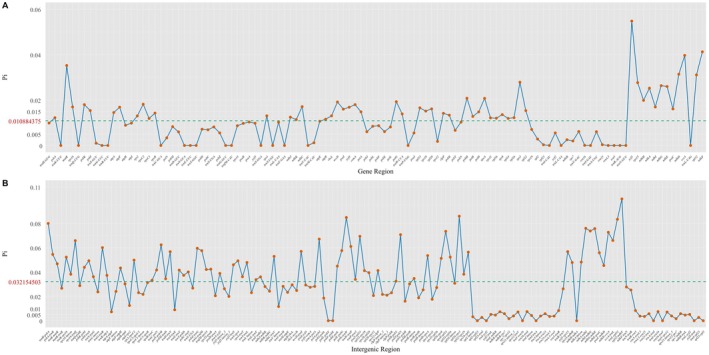
Nucleotide diversity (Pi) values across the chloroplast genomes of 23 *Ranunculus* species. (A) Pi values in gene regions. (B) Pi values in intergenic regions. The horizontal axis represents different genomic regions, and the vertical axis indicates the nucleotide diversity (Pi). The dashed line in each panel marks the average Pi value for the respective region (0.0109 for genes, 0.0232 for intergenic regions).

Certain chloroplast regions are more prone to mutation, making them useful for species identification, population genetics, plant barcoding, and phylogenetic inference (Daniell et al. 2016; Dong et al. 2015; Shaw et al. 2007). Nucleotide diversity analysis showed that intergenic spacers such as *rpl32–ndhF* (π = 0.10015), *rpl16–rps3* (π = 0.08595), and *petG–trnW* (π = 0.08489) were highly polymorphic, exceeding the variability of the standard barcode *trnH–psbA* (π = 0.07998). Within coding regions, *ycf1* (π = 0.05478), *ndhF* (π = 0.04124), and *ccsA* (π = 0.03966) were more polymorphic (Table 3, Figure 7; Table S10). *ycf1* and *ndhF* are also commonly employed in phylogenetic studies, and *ycf1* has also been proposed as a core DNA barcode for land plants (Dong et al. 2015). These loci therefore represent promising candidates for taxon‐specific identification, population genetic and phylogenetic studies in *Ranunculus*. Notably, *ycf1* and *ndhF* have also proven effective in metabarcoding of *Paeonia* species (Yang et al. 2022).

**TABLE 3:** Identified polymorphic loci based on comparative genomic analysis of *Ranunculus* species, ten each from non‐coding and protein coding regions.

Serial number	Region	Nucleotide diversity	Number of substitutions	Number of indels	Region length	Alignment length
1	*ycf1*	0.05478	929	23	5255	5638
2	*ndhF*	0.04124	280	5	2193	2246
3	*ccsA*	0.03966	119	4	957	983
4	*matK*	0.03521	165	3	1512	1539
5	*ndhD*	0.03139	146	2	1530	1542
6	*rpl32*	0.03108	16	0	162	162
7	*rps3*	0.02785	62	1	654	657
8	*rps15*	0.02768	22	0	273	273
9	*ndhG*	0.02634	43	1	534	539
10	*ndhE*	0.02598	25	1	306	312
11	*rpl32‐ndhF*	0.10015	137	93	702	1148
12	*rpl16‐rps3*	0.08595	26	15	157	206
13	*petG‐trnW*	0.08489	25	12	138	186
14	*trnL‐rpl32*	0.08339	121	58	617	955
15	*trnH‐psbA*	0.07998	21	40	196	402
16	*ndhA‐ndhI*	0.07597	19	2	73	82
17	*ndhG‐ndhE*	0.07573	43	15	231	301
18	*ndhI‐ndhG*	0.07376	67	24	343	421
19	*rps8‐rpl14*	0.0735	26	27	230	380
20	*ndhD‐ccsA*	0.07271	21	19	169	221

Notably, a comparative analysis between our assembled *R. arvensis* chloroplast genome (sampled from Pakistan) and a recently released accession (GenBank: PV621859) originating from a distinct geographic population (China) revealed 100% sequence identity with zero single nucleotide polymorphisms (SNPs). This absolute lack of intraspecific variation across such vast geographic distances further underscores the extreme evolutionary conservation of the *R. arvensis* cp genome.

### 3.8 Phylogenetic Analysis

**FIGURE 8:**
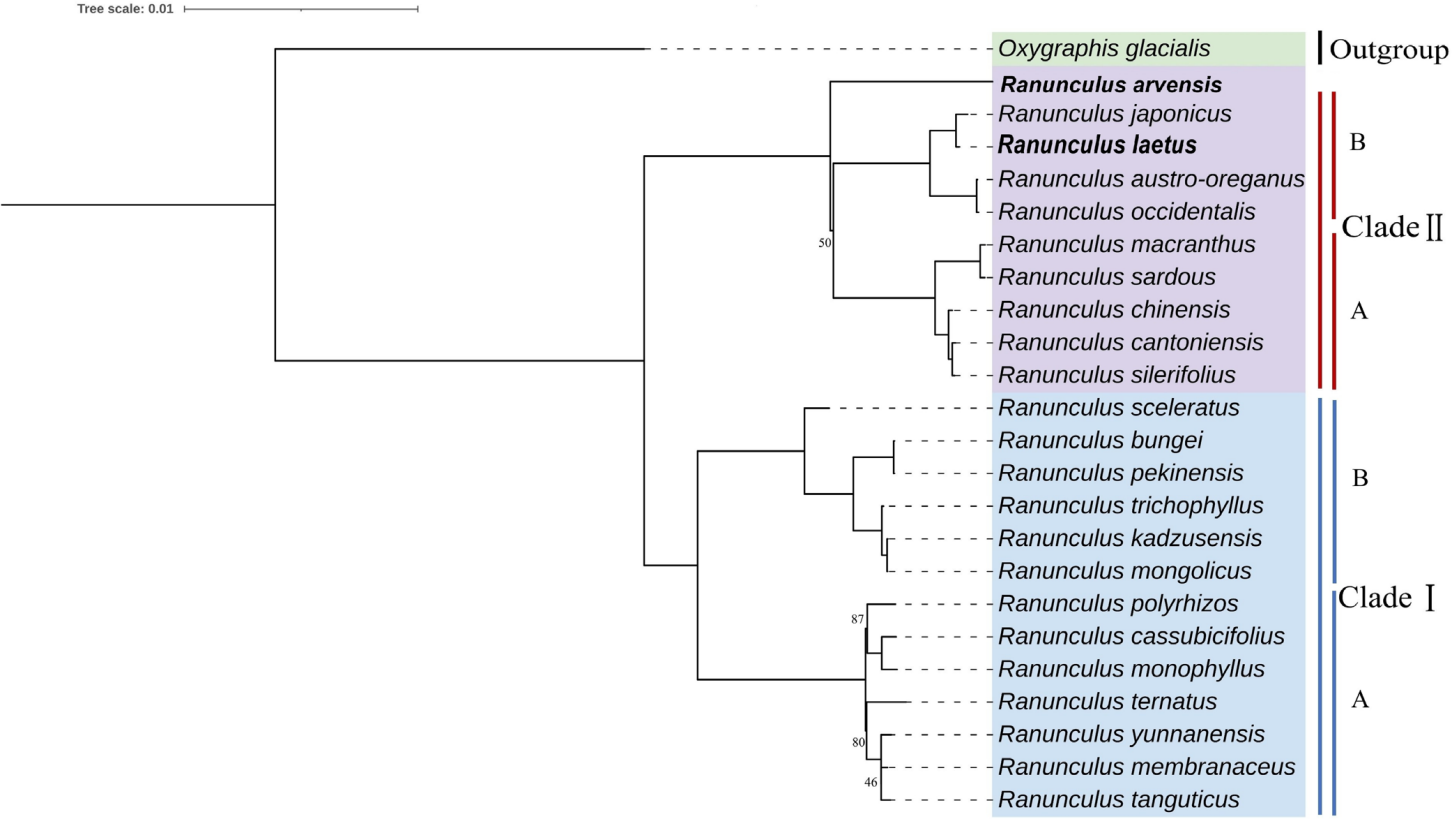
Phylogenetic tree of 23 *Ranunculus* species inferred from complete chloroplast genome sequences. The two species assembled in this study are highlighted in bold. The tree is rooted with *Oxygraphis glacialis* as the outgroup. All branches are drawn to scale (scale bar = 0.01 substitutions per site), and the two major clades (Clade I, Clade II) identified in this study are indicated. Bootstrap values equal to 100 are not shown for clarity.

The phylogenetic analysis, with *Oxygraphis glacialis* (NC_041538) as the outgroup, resolved *Ranunculus* into two strongly supported major sister clades (BS = 100%)—each further subdivided into two well‐supported subclades (Subclade I‐A and I‐B; Subclade II‐A and II‐B)—providing a stable framework for interpreting evolutionary and ecological patterns across the sampled taxa (Figure 8).

Clade I comprised a large assemblage made up of several well‐supported subclades. One lineage (Subclade I‐A) contained *Ranunculus yunnanensis*, *Ranunculus tanguticus*, and *R. membranaceus*, with *R. tanguticus* and *R. membranaceus* forming a weakly supported sister pair (BS = 46%) that together grouped with *Ranunculus ternatus* (BS = 100%). A second subclade included *R. polyrhizos*, sister to a strongly supported pair of *Ranunculus monophyllus* and *R. cassubicifolius* (BS = 100%); these subclades were united with maximum support (BS = 100%). Another lineage (Subclade I‐B) within Clade I grouped the hydrosubshrubs *R. trichophyllus* , *R. mongolicus*, and *R. kadzusensis* (BS = 100%), and this assemblage was allied to *R. bungei* (including *Ranunculus pekinensis* as synonym) (BS = 100%); the whole was sister to *R. sceleratus* with full support.

Clade II had a more restricted taxonomic composition but similarly well‐supported internal structure. Within Subclade II‐A, *Ranunculus silerifolius* var. *silerifolius*, *R. cantoniensis* , and *R. chinensis* formed a strongly supported clade (BS = 100%) sister to *Ranunculus sardous* and *Ranunculus macranthus* (BS = 100%). Within Subclade II‐B, North American taxa ( *R. occidentalis* , *Ranunculus austro‐oreganus* ) formed a strongly supported sister pair (BS = 100%) that grouped with Asian species (*R. laetus*, *R. japonicus* ) with maximum support (BS = 100%). Notably, the placement of *R. arvensis* at the base of the entire Clade II received only moderate support (BS = 50%), indicating either unresolved deeper relationships, rapid diversification, or historical complexity such as hybridization or incomplete lineage sorting. Rather than clear regional structuring, Clade II displays mixed clustering of East Asian and North American taxa, consistent with transcontinental affinities documented in other Ranunculaceae or long‐distance dispersal events.

The habitat diversity in Clade I (including East Asian hydrosubshrubs and terrestrial taxa) contrasts with Clade II, which combines East Asian and North American species, likely reflecting historical long‐distance dispersal events and different biogeographical processes. Paun et al. highlighted the Mediterranean and alpine zones as secondary diversification centers with strong geographical structure, while Emadzade et al. (2011) reported clear geographic partitioning of alpine lineages between Central Asia and North America (Paun et al. 2005). In contrast, the present tree suggests East Asian diversification alongside transcontinental assemblages, indicating different evolutionary dynamics in the groups sampled here compared to Mediterranean and high mountain systems.

Although cytogenetic data were not analyzed in this study, both Paun et al. and Emadzade et al. documented frequent polyploidy and reticulate evolution in *Ranunculus* (Emadzade et al. 2011; Paun et al. 2005). Polyploidy may underlie the moderate bootstrap support values observed in our tree (e.g., 46% for the *R. tanguticus*–*R. membranaceus* relationship and 50% for *R. arvensis* placement in Clade II), consistent with reticulate signals reported in other *Ranunculus* complexes. For transcontinental relationships, such as the pairing of *R. occidentalis* with *R. laetus* and *R. japonicus* , polyploidy and hybridization may have facilitated both lineage differentiation and subsequent reproductive isolation, as suggested by Emadzade et al. (2011).

Subsequent analyses using individual gene datasets largely recovered the same overall grouping of species into these major clades, indicating a consistent higher‐level phylogenetic signal. However, the relationships among species within each clade were not congruent with those inferred from the main concatenated dataset and varied significantly across individual gene trees.

To explicitly validate this robustness and test the utility of specific DNA barcodes, we conducted targeted comparative analyses using the 20 highly variable regions (10 CDS and 10 IGS) identified in this study. Consistent with the broader single‐gene analyses, we observed notable topological discordance. For most individual CDS, the broad division of species was recovered, but internal relationships conflicted with the main phylogeny and exhibited low bootstrap support. Comparatively, genes such as *matK*, *ycf1*, and *ccsA* performed better than others. These markers successfully recovered the broad divergence of the two major clades (Clade I and Clade II) and resolved the internal relationships within Subclade I‐B and most of Clade II consistently with the main cp genome phylogeny (with the exception of the exact placement of *R. arvensis* ).

However, their limitations within certain specific lineages are pronounced. Within Subclade I‐A, the topologies generated by *matK*, *ycf1*, and *ccsA* were largely discordant with the whole‐cp genome tree, and the bootstrap support values for critical backbone nodes within this subclade were frequently extremely low or even 0%. Other genes performed even worse; for instance, markers like *rpl32* and *rps15* failed to even accurately reflect the basic clade divisions. Trees based on individual IGS regions showed even greater discordance, differing substantially from the complete cp genome tree. Given that individual loci proved insufficient for robust species‐level resolution, we synthesized the genetic signal of the 10 highly variable CDS using both concatenation and consensus approaches. Remarkably, both the concatenated 10‐CDS tree and the majority‐rule consensus tree yielded congruent topologies that were consistent with the complete cp genome phylogeny, with only minor topological variance occurring within the internal branches of Clade I‐A (Figures S3–S24). This localized discrepancy underscores the fact that while a combination of highly informative genes can recover the broad evolutionary history, complete plastome sequences remain essential for fully resolving rapid diversification events within specific sub‐lineages.

The stark contrast between single‐locus and multi‐locus phylogenies highlights the profound evolutionary complexity of the genus *Ranunculus*. This discordance likely reflects a combination of limited parsimony‐informative sites within short sequences, heterogeneity in evolutionary rates across different plastid regions, and the rapid radiation of the genus (Emadzade et al. 2011; Hörandl et al. 2005). Furthermore, potential biological processes such as incomplete lineage sorting (ILS) and historical hybridization—which are common in *Ranunculus*—can lead to localized gene tree conflicts (Paun et al. 2005; Wang et al. 2009). We acknowledge that the concatenation approach alone may obscure underlying gene tree conflicts by enforcing a single topology; however, by computing a consensus tree alongside concatenation, we successfully cross‐validated the results, increased the phylogenetic signal‐to‐noise ratio, and effectively buffered against the stochastic errors of individual genes (Gitzendanner et al. 2018). Collectively, these results suggest that while the major clade structure is robust, the finer‐scale relationships within clades remain less certain and should be interpreted with caution. Additionally, these findings demonstrate that traditional DNA barcoding relying on a single or a few loci is largely inadequate for resolving closely related *Ranunculus* species (CBOL Plant Working Group 2009; Hollingsworth et al. 2011). Instead, species identification and phylogenetic reconstruction in this complex lineage must increasingly rely on whole‐cp genome sequences or, at a minimum, multi‐gene datasets that aggregate robust signals from multiple highly variable regions (Li et al. 2015).

## 4 Conclusion

This study presents the first complete chloroplast genome of *R. laetus*, together with an additional cp genome assembly of *R. arvensis* (from a Pakistani population) that complements the recently released GenBank accession PV621859, providing comprehensive comparative analysis with 21 congeners. The genomes exhibited conserved quadripartite structures with no major rearrangements, though lineage‐specific variations in *atpH* position were detected in two species. Crucially, advanced evolutionary analyses utilizing multiple analytical frameworks (BUSTED, MEME, and FUBAR) uncovered a dynamic selective landscape: while the photosynthetic apparatus remained highly conserved, robust signatures of episodic and pervasive positive selection were detected across ~34.6% of the annotated plastid genes, particularly highlighting *matK* and *ycf1* as critical adaptive hotspots. Nucleotide diversity analysis identified highly variable regions (*rpl32–ndhF*, *ycf1* and *ndhF*) exceeding standard barcodes in discriminatory power. Phylogenetic reconstruction incorporating both whole‐cp genome and single‐gene topologies strongly supported *Ranunculus* monophyly and resolved two major clades reflecting biogeographic patterns.

These genomic resources provide a foundation for future studies in *Ranunculus* systematics, DNA barcoding, and population genetics. We recommend expanded taxonomic sampling across the genus's ~1760 species to further elucidate evolutionary patterns and biogeographic history within Ranunculaceae.

## Author Contributions

Hui Li: conceptualization, data curation, formal analysis, investigation, writing – original draft. Siyue Zhang: formal analysis, writing – original draft, writing – review and editing. Abdullah: data curation, conceptualization, formal analysis, methodology, visualization, writing – original draft, writing – review and editing. Sayed Afzal Shah: resources, validation. Yuhua Huang: data curation, formal analysis, Jingjing Jia: formal analysis. Liya Guo: investigation. Ying Cui: conceptualization, writing – review and editing, Jiahui Sun: conceptualization, writing – review and editing. Parviz Heidari: conceptualization, investigation, methodology, writing – review and editing. Xiaoxuan Tian: conceptualization, investigation, resources, project administration, supervision, writing – review and editing.

## Funding

This study was supported by Key project at central government level: The ability establishment of sustainable use for valuable Chinese medicine resources (National Administration of Traditional Chinese Medicine (NATCM), 2060302).

## Conflicts of Interest

The authors declare no conflicts of interest.

## Supporting information


**Figure S1:** Sequencing depth and coverage for *Ranunculus arvensis*.
**Figure S2:** Sequencing depth and coverage for *Ranunculus laetus*.
**Figure S3:** Phylogenetic tree of 23 *Ranunculus* species based on 10 concatenated protein‐coding genes.
**Figure S4:** Consensus tree of 23 *Ranunculus* species derived from 10 individual protein‐coding genes.
**Figure S5:** Phylogenetic tree of 23 *Ranunculus* species based on the *ccsA* gene.
**Figure S6:** Phylogenetic tree of 23 *Ranunculus* species based on the *matK* gene.
**Figure S7:** Phylogenetic tree of 23 *Ranunculus* species based on the *ndhD* gene.
**Figure S8:** Phylogenetic tree of 23 *Ranunculus* species based on the *ndhE* gene.
**Figure S9:** Phylogenetic tree of 23 *Ranunculus* species based on the *ndhF* gene.
**Figure S10:** Phylogenetic tree of 23 *Ranunculus* species based on the *ndhG* gene.
**Figure S11:** Phylogenetic tree of 23 *Ranunculus* species based on the *rpl32* gene.
**Figure S12:** Phylogenetic tree of 23 *Ranunculus* species based on the *rps3* gene.
**Figure S13:** Phylogenetic tree of 23 *Ranunculus* species based on the *rps15* gene.
**Figure S14:** Phylogenetic tree of 23 *Ranunculus* species based on the *ycf1* gene.
**Figure S15:** Phylogenetic tree of 23 *Ranunculus* species based on the *ccsA‐ndhD* IGS regions.
**Figure S16:** Phylogenetic tree of 23 *Ranunculus* species based on the *ndhA‐ndhI* IGS regions.
**Figure S17:** Phylogenetic tree of 23 *Ranunculus* species based on the *ndhE‐ndhG* IGS regions.
**Figure S18:** Phylogenetic tree of 23 *Ranunculus* species based on the *ndhF‐rpl32* IGS regions.
**Figure S19:** Phylogenetic tree of 23 *Ranunculus* species based on the *ndhG‐ndhI* IGS regions.
**Figure S20:** Phylogenetic tree of 23 *Ranunculus* species based on the *petG‐trnW* IGS regions.
**Figure S21:** Phylogenetic tree of 23 *Ranunculus* species based on the *psbA‐trnH* IGS regions.
**Figure S22:** Phylogenetic tree of 23 *Ranunculus* species based on the *rpl14‐rps8* IGS regions.
**Figure S23:** Phylogenetic tree of 23 *Ranunculus* species based on the *rpl16‐rps3* IGS regions.
**Figure S24:** Phylogenetic tree of 23 *Ranunculus* species based on the *rpl32‐trnL* IGS regions.

**Table S1:** Characteristics and best‐fit models of phylogenetic datasets.

**Table S2:** Intron lengths in intron‐containing genes (CDS and tRNA) across *Ranunculus* species.

**Table S3:** Relative synonymous codon usage analysis of the species of genus *Ranunculus*.

**Table S4:** Amino acids frequency among 23 *Ranunculus* species.

**Table S5:** Comparison of SSRs numbers and their types among *Ranunculus* species.

**Table S6:** Simple sequence repeats motifs and their types in species of *Ranunculus*.

**Table S7:** Substitutions analysis of three main reagions of chloroplast genome in *Ranunculus*.

**Table S8:** Selection pressure analysis using MEME, BUSTED, and FUBAR models.

**Table S9:** Detailed selection pressure analysis results using MEME.

**Table S10:** Nucleotide diversity (π) of gene regions and intergenic regions.

## Data Availability

The complete chloroplast genome sequence data for *R. arvensis* and *R. laetus* were submitted to NCBI under GenBank accession numbers PV364608 and PV364609, respectively. The raw sequencing data can be accessed through BioProject PRJNA1276266.
