# Supplementary material for: Complete Chloroplast Genomes of Ranunculus arvensis and Ranunculus laetus: Comparative Analysis and Phylogenetic Insights
Source: Ecol Evol. 2026 Apr 29;16(5):e73559. doi: 10.1002/ece3.73559 (PMC13128338; doi:10.1002/ece3.73559)
Supplement: Supplementary file 1 — Figure S1: Sequencing depth and coverage for Ranunculus arvensis. Figure S2: Sequencing depth and coverage for Ranunculus laetus. Figure S3: Phylogenetic tree of 23 Ranunculus species based on 10 concatenated protein‐coding genes. Figure S4: Consensus tree of 23 Ranunculus species derived from 10 individual protein‐coding genes. Figure S5: Phylogenetic tree of 23 Ranunculus species based on the ccsA gene. Figure S6: Phylogenetic tree of 23 Ranunculus species based on the matK gene. Figure S7: Phylogenetic tree of 23 Ranunculus species based on the ndhD gene. Figure S8: Phylogenetic tree of 23 Ranunculus species based on the ndhE gene. Figure S9: Phylogenetic tree of 23 Ranunculus species based on the ndhF gene. Figure S10: Phylogenetic tree of 23 Ranunculus species based on the ndhG gene. Figure S11: Phylogenetic tree of 23 Ranunculus species based on the rpl32 gene. Figure S12: Phylogenetic tree of 23 Ranunculus species based on the rps3 gene. Figure S13: Phylogenetic tree of 23 Ranunculus species based on the rps15 gene. Figure S14: Phylogenetic tree of 23 Ranunculus species based on the ycf1 gene. Figure S15: Phylogenetic tree of 23 Ranunculus species based on the ccsA‐ndhD IGS regions. Figure S16: Phylogenetic tree of 23 Ranunculus species based on the ndhA‐ndhI IGS regions. Figure S17: Phylogenetic tree of 23 Ranunculus species based on the ndhE‐ndhG IGS regions. Figure S18: Phylogenetic tree of 23 Ranunculus species based on the ndhF‐rpl32 IGS regions. Figure S19: Phylogenetic tree of 23 Ranunculus species based on the ndhG‐ndhI IGS regions. Figure S20: Phylogenetic tree of 23 Ranunculus species based on the petG‐trnW IGS regions. Figure S21: Phylogenetic tree of 23 Ranunculus species based on the psbA‐trnH IGS regions. Figure S22: Phylogenetic tree of 23 Ranunculus species based on the rpl14‐rps8 IGS regions. Figure S23: Phylogenetic tree of 23 Ranunculus species based on the rpl16‐rps3 IGS regions. Figure S24: Phylogenetic tree of 23 Ranunculus species based [file ECE3-16-e73559-s001.docx]

**Supplementary figures**


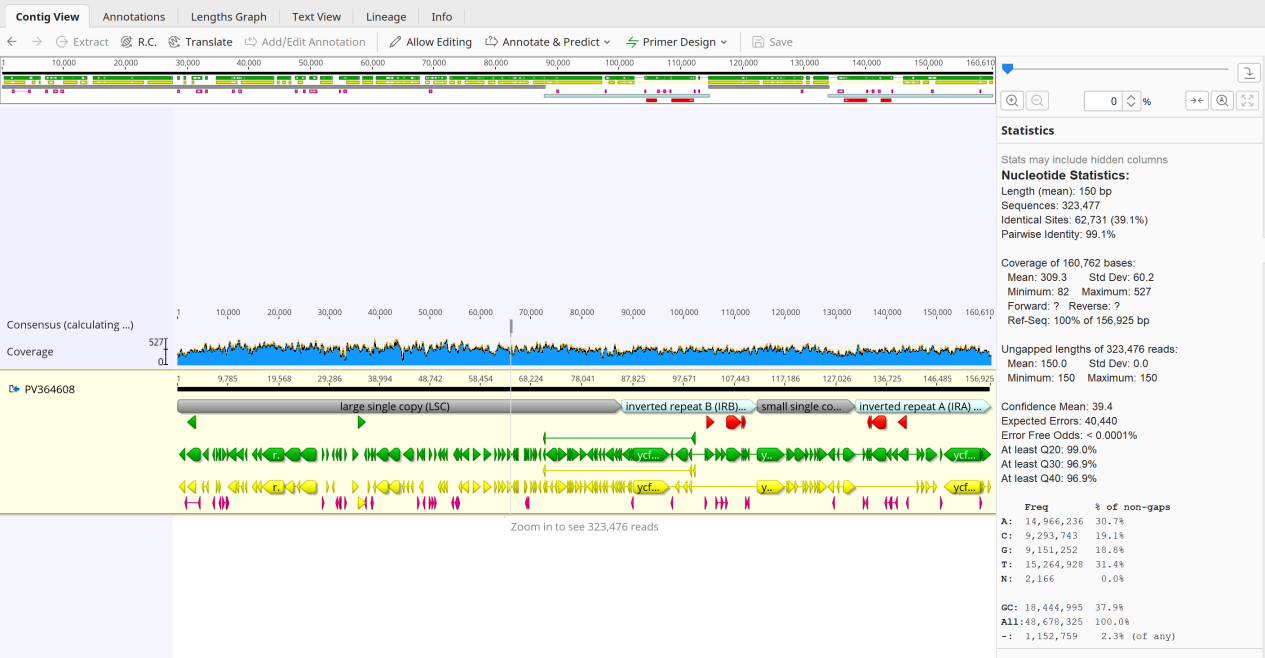


Figure S1. Sequencing Depth and Coverage for Ranunculus arvensis.


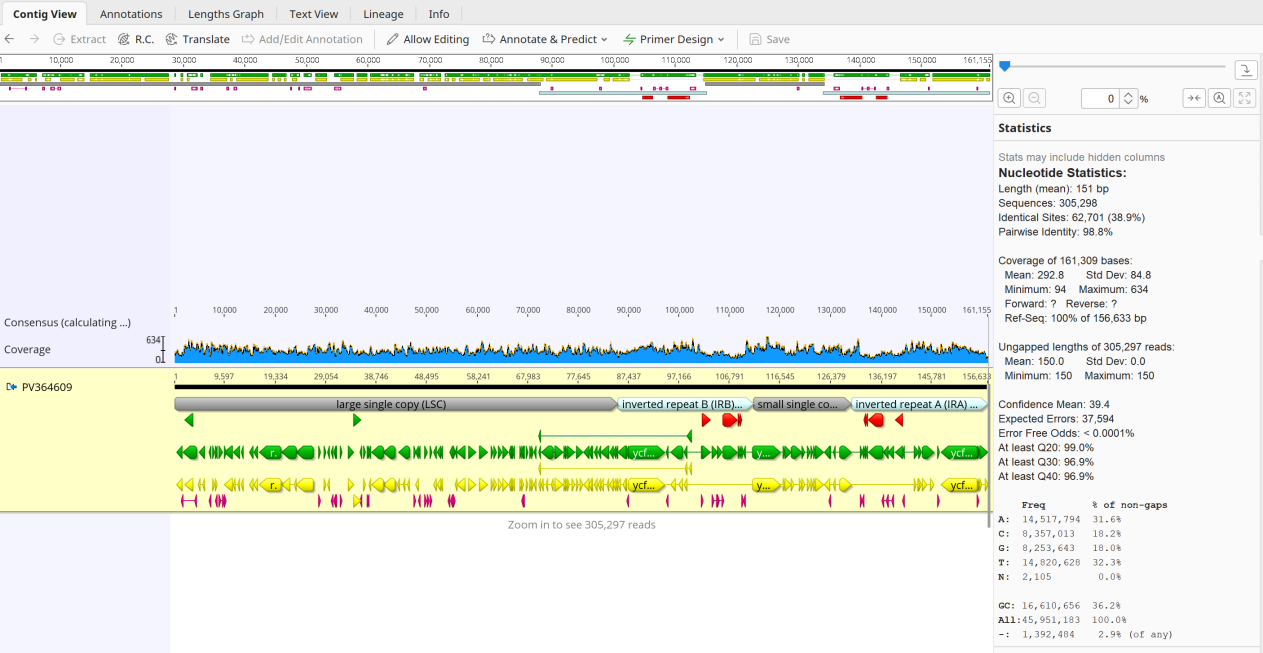


Figure S2. Sequencing Depth and Coverage for *Ranunculus laetus.*


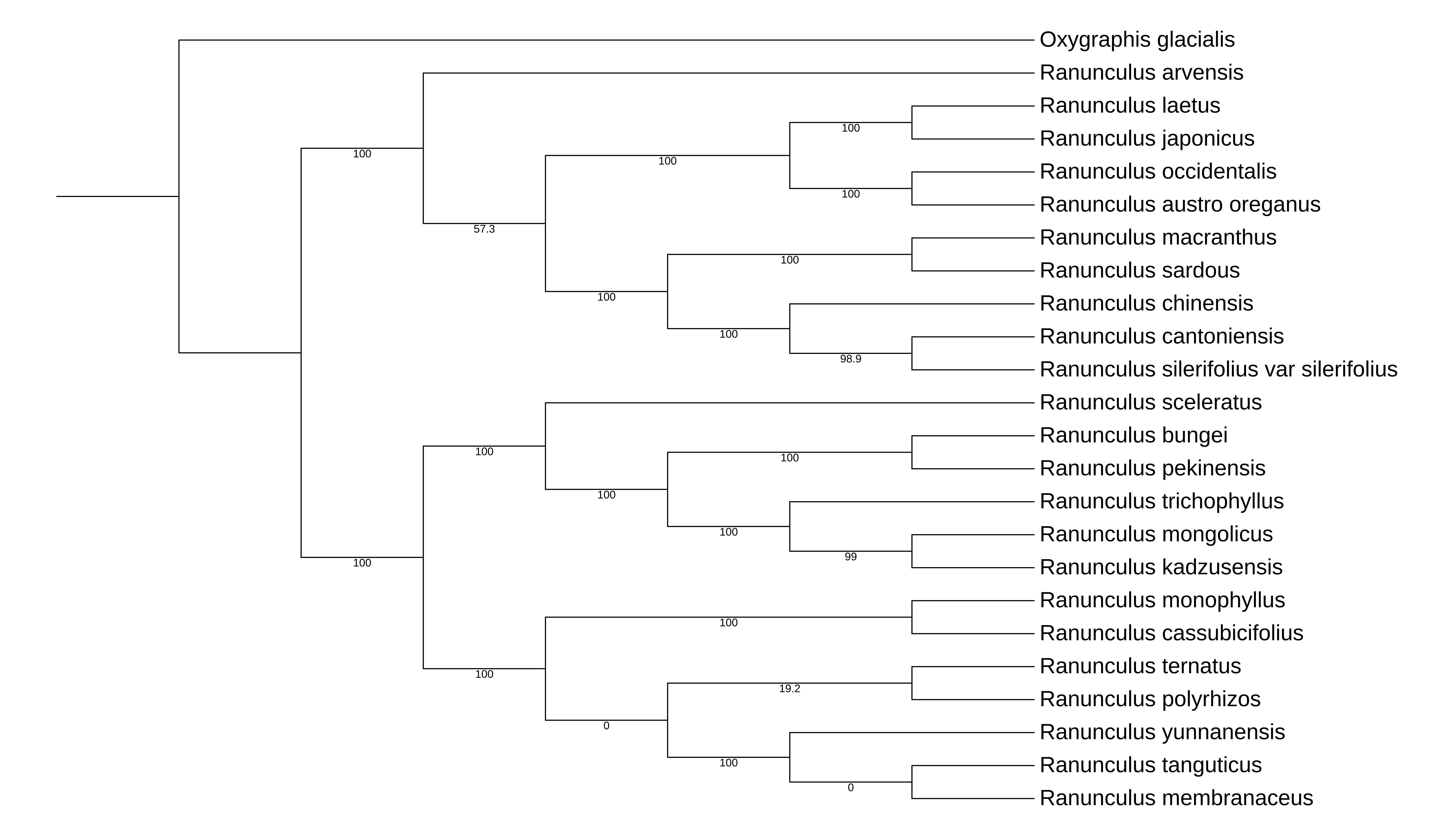


Figure S3. Phylogenetic tree of 23 *Ranunculus* species based on 10 concatenated protein-coding genes.


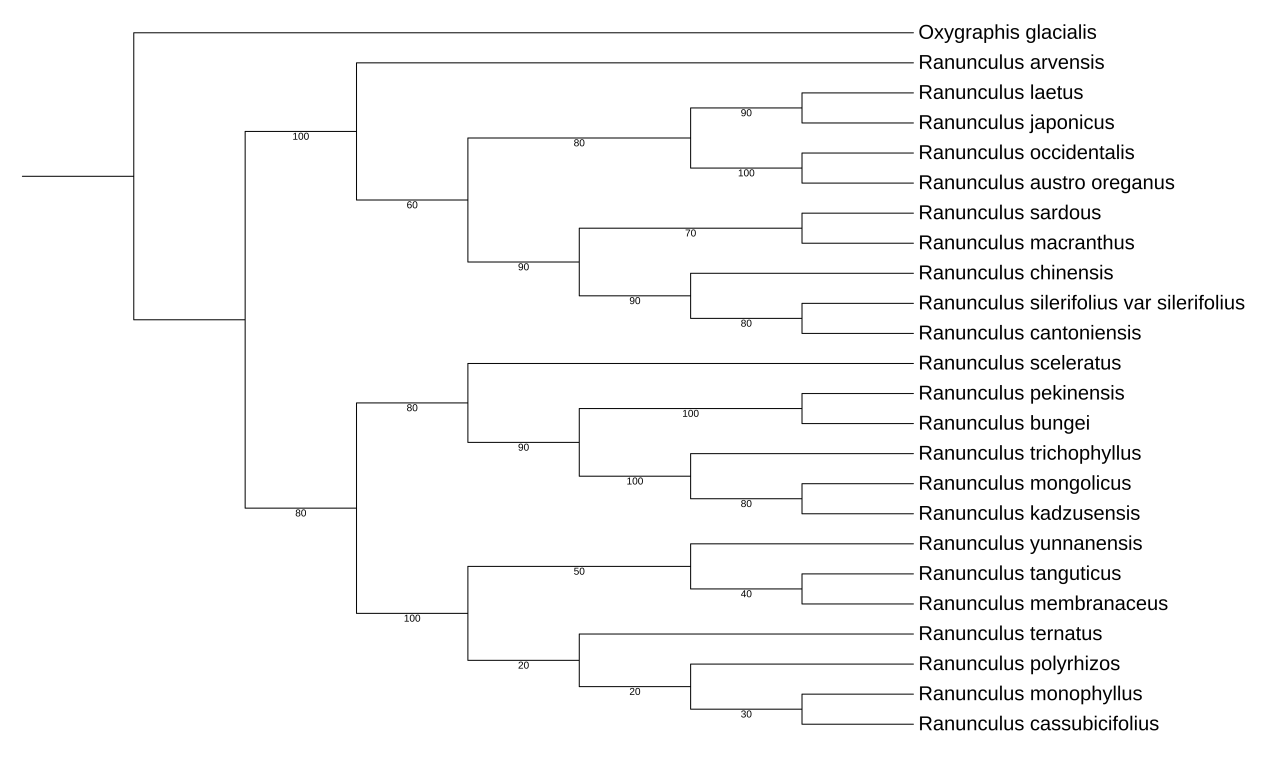


Figure S4. Consensus tree of 23 *Ranunculus* speciesderived from 10 individual protein-coding gene trees.


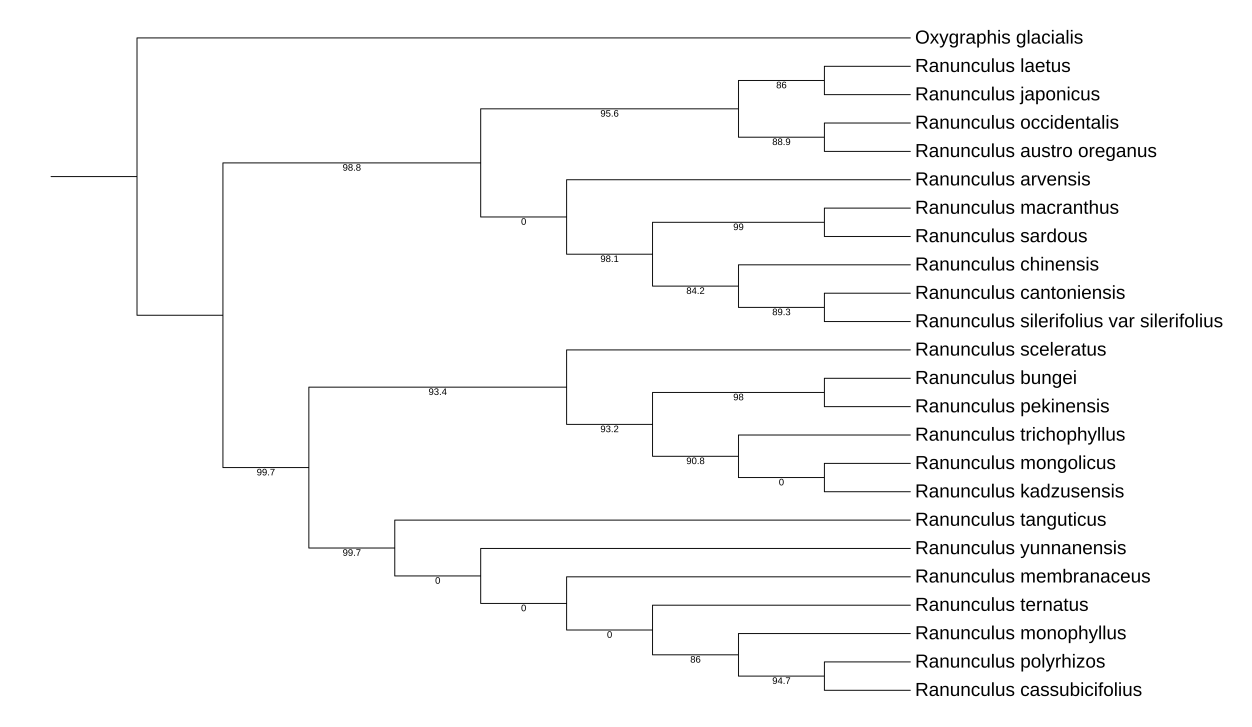


Figure S5. Phylogenetic tree of 23 *Ranunculus* species based on the *ccsA* gene.


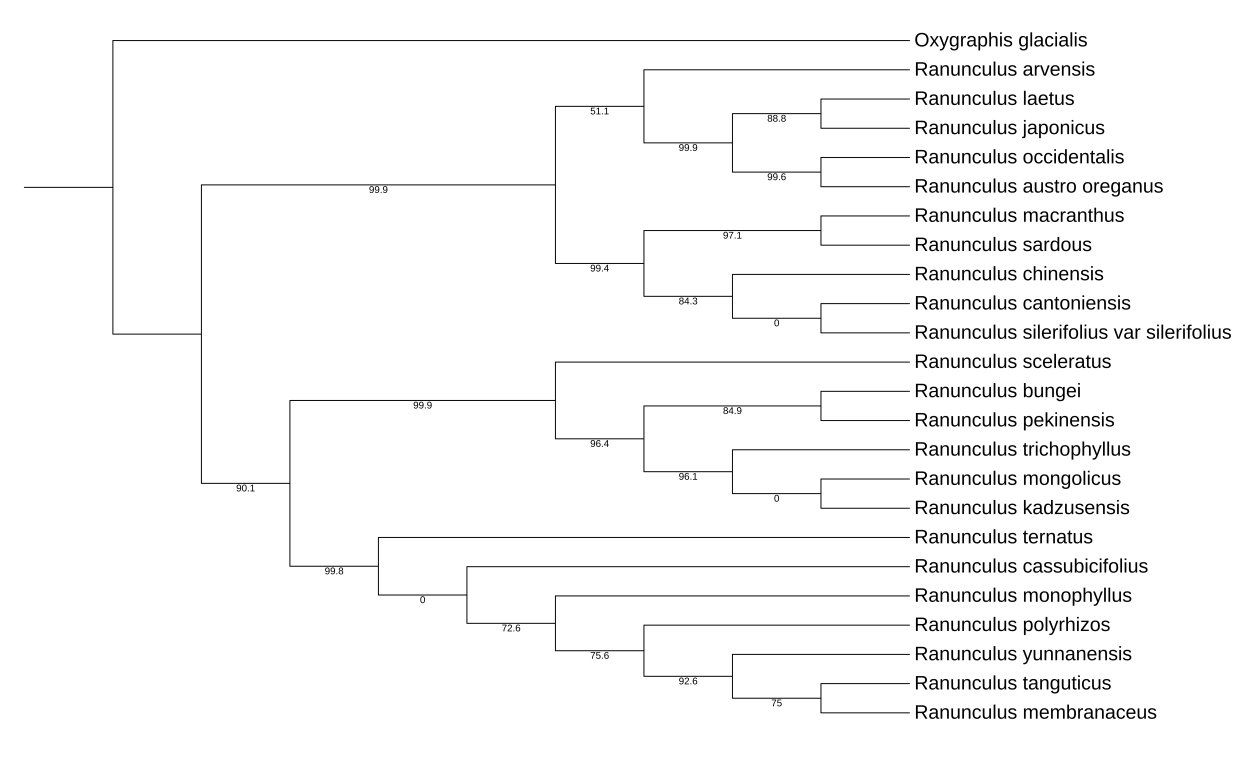


Figure S6. Phylogenetic tree of 23 *Ranunculus* species based on the *matK* gene.


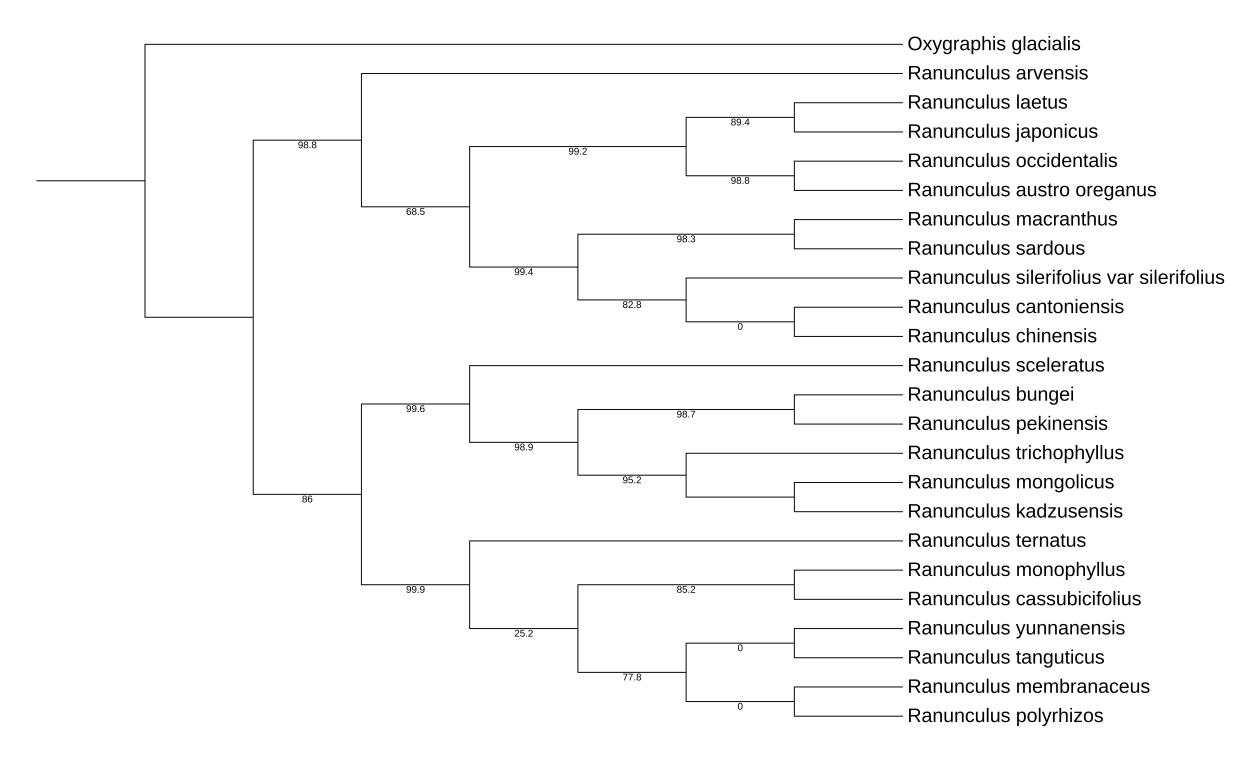


Figure S7. Phylogenetic tree of 23 *Ranunculus* species based on the *ndhD* gene.


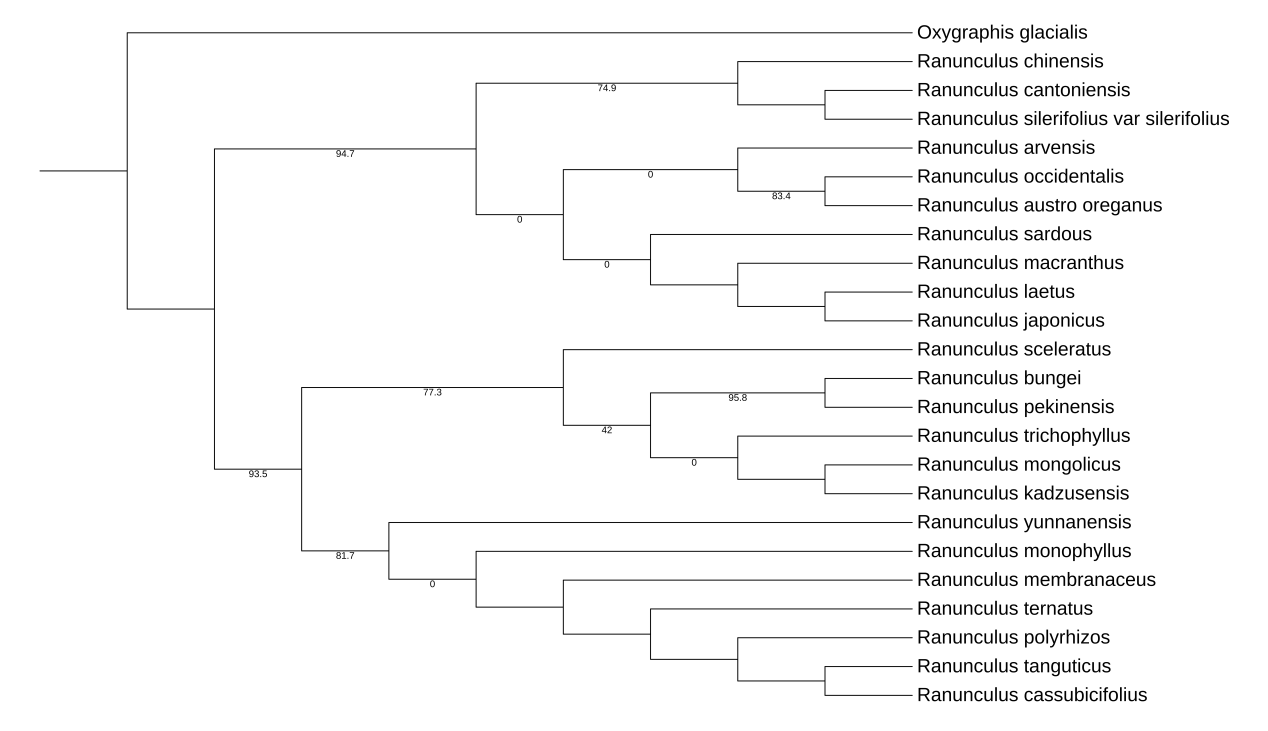


Figure S8. Phylogenetic tree of 23 *Ranunculus* species based on the *ndhE* gene.


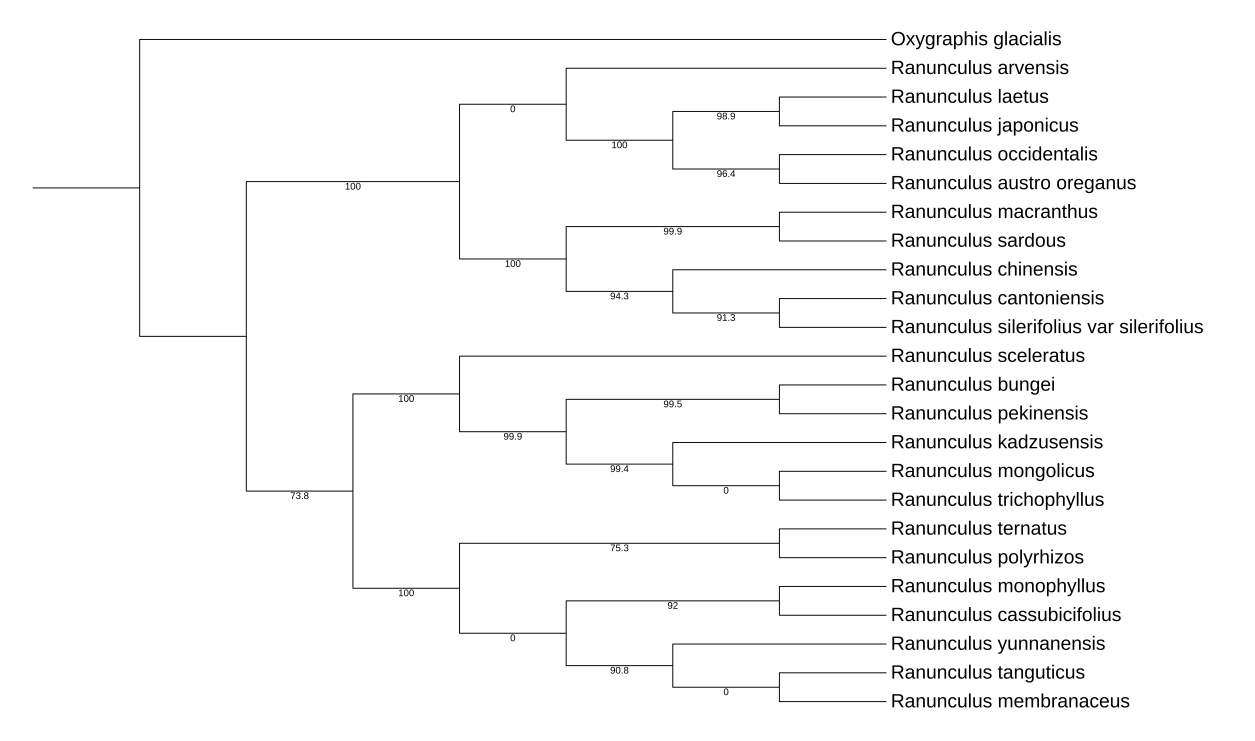


Figure S9. Phylogenetic tree of 23 *Ranunculus* species based on the *ndhF* gene.


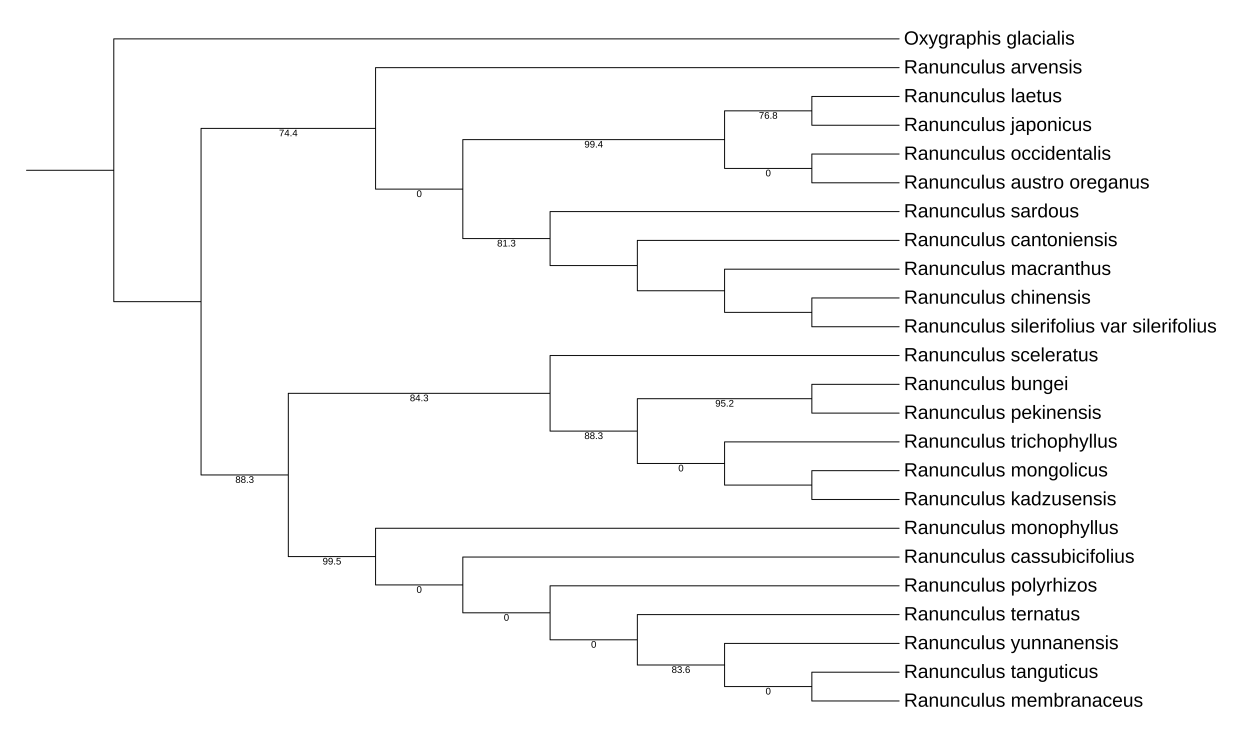


Figure S10. Phylogenetic tree of 23 *Ranunculus* species based on the *ndhG* gene.


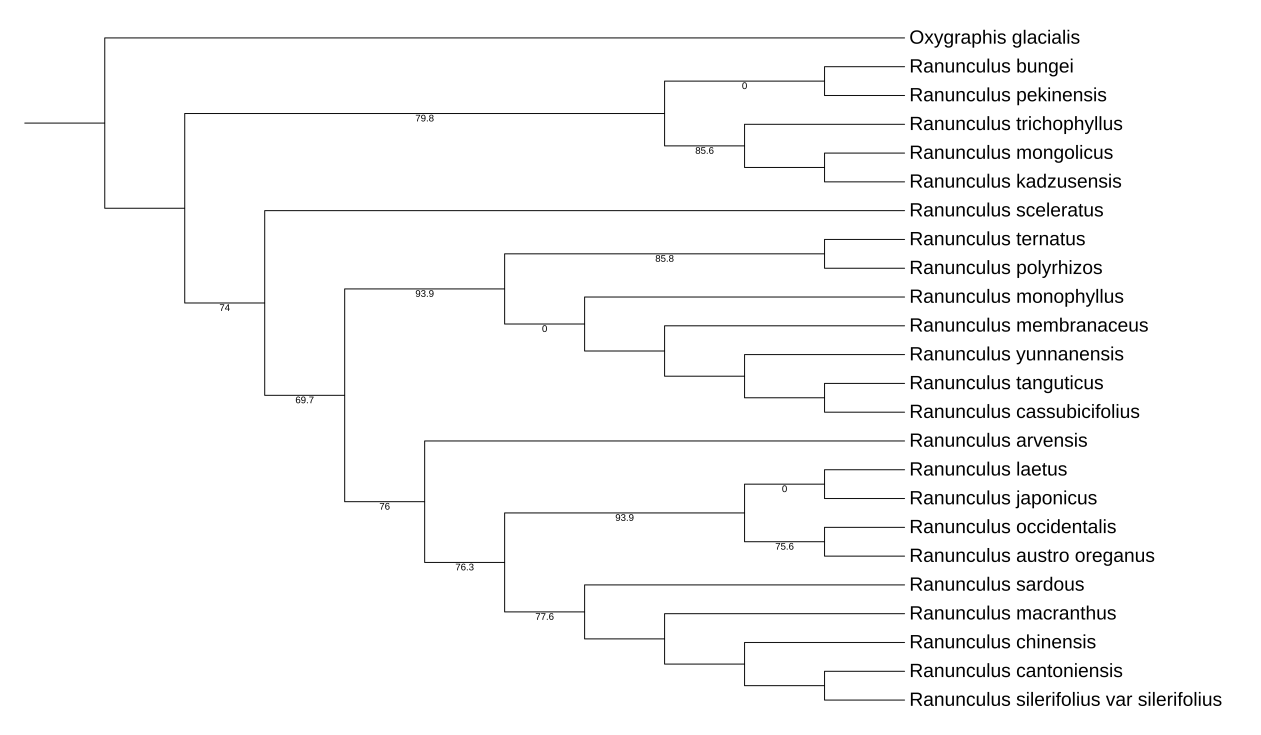


Figure S11. Phylogenetic tree of 23 *Ranunculus* species based on the *rpl32* gene.


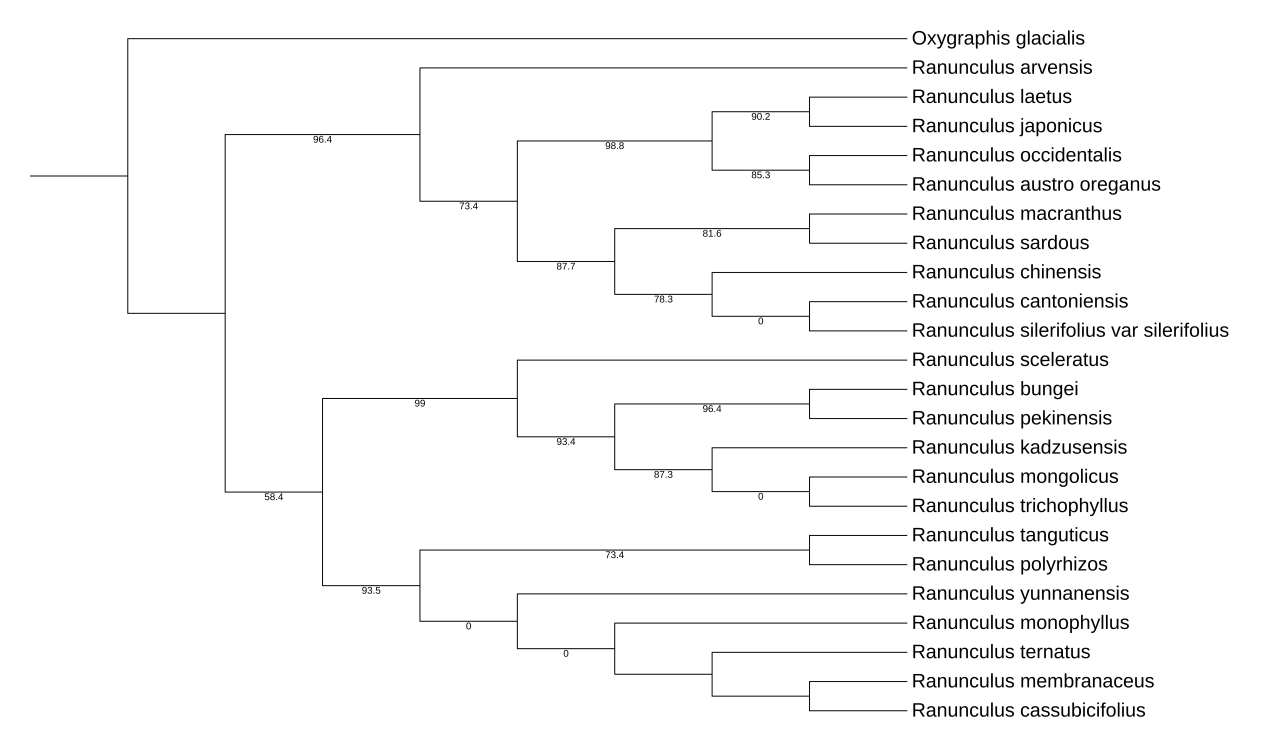


Figure S12. Phylogenetic tree of 23 *Ranunculus* species based on the *rps3* gene.


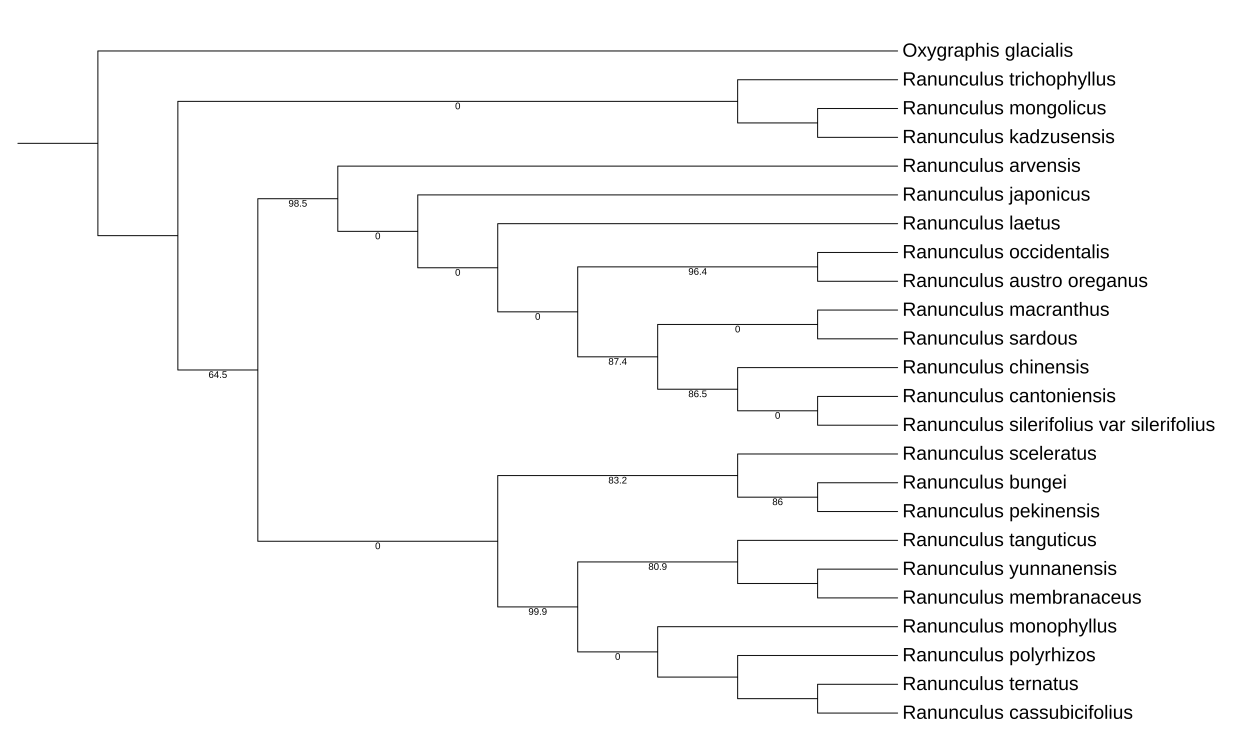


Figure S13. Phylogenetic tree of 23 *Ranunculus* species based on the *rps15* gene.


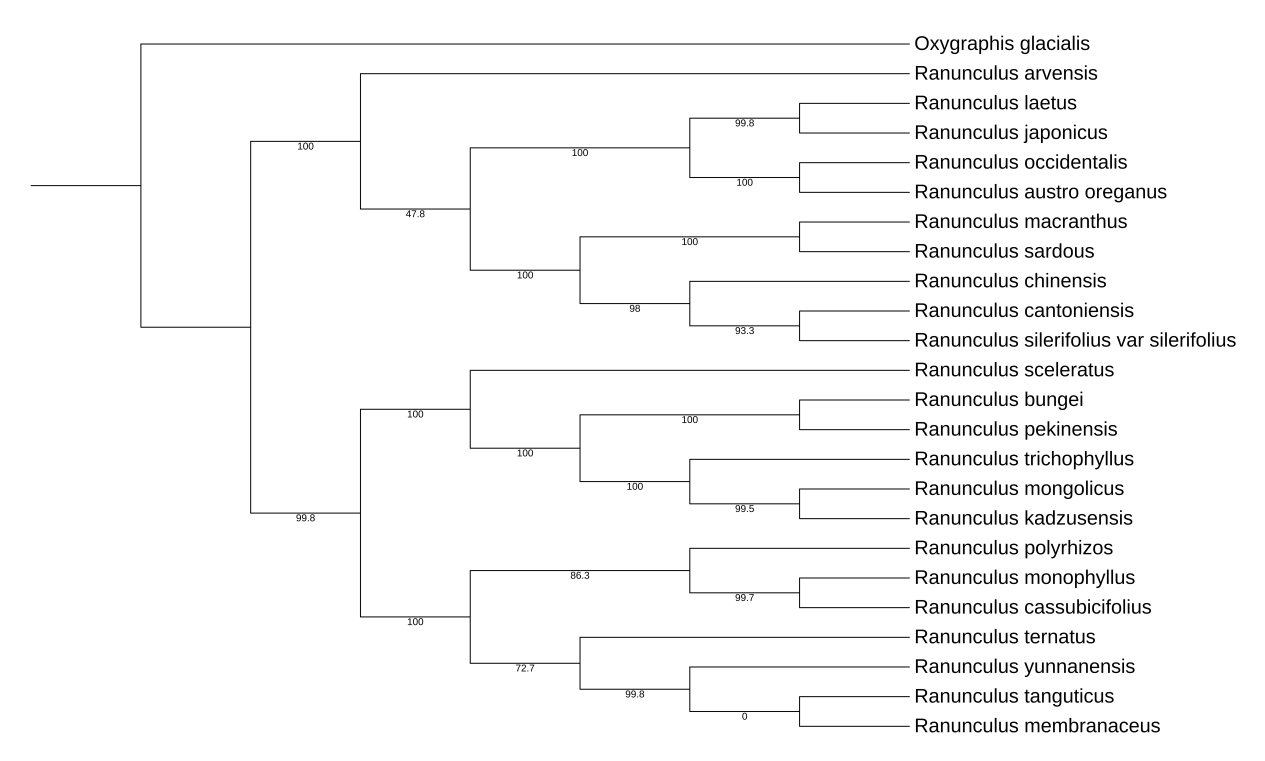


Figure S14. Phylogenetic tree of 23 *Ranunculus* species based on the *ycf1* gene.


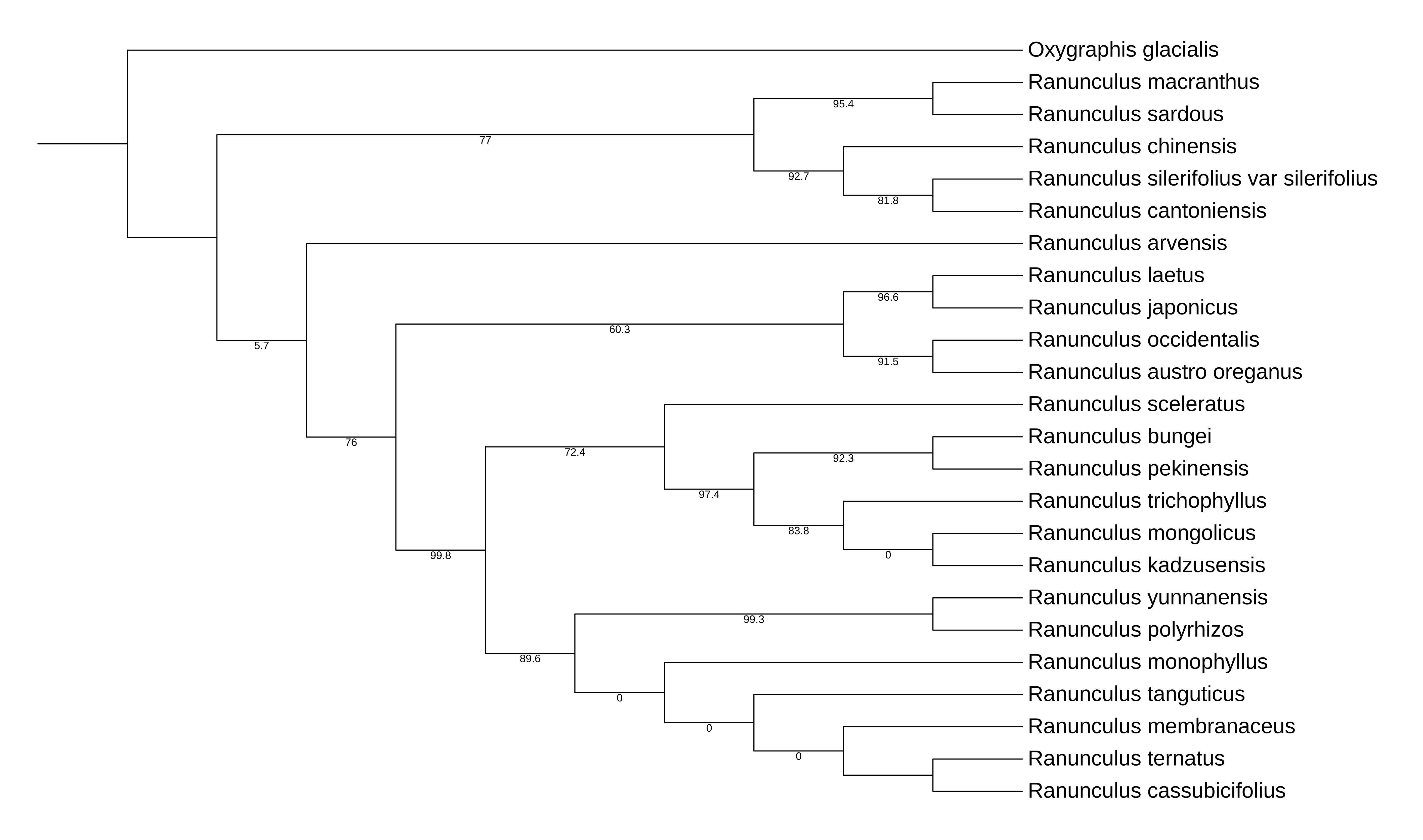


Figure S15. Phylogenetic tree of 23 *Ranunculus* species based on the *ccsA-ndhD* IGS regions.


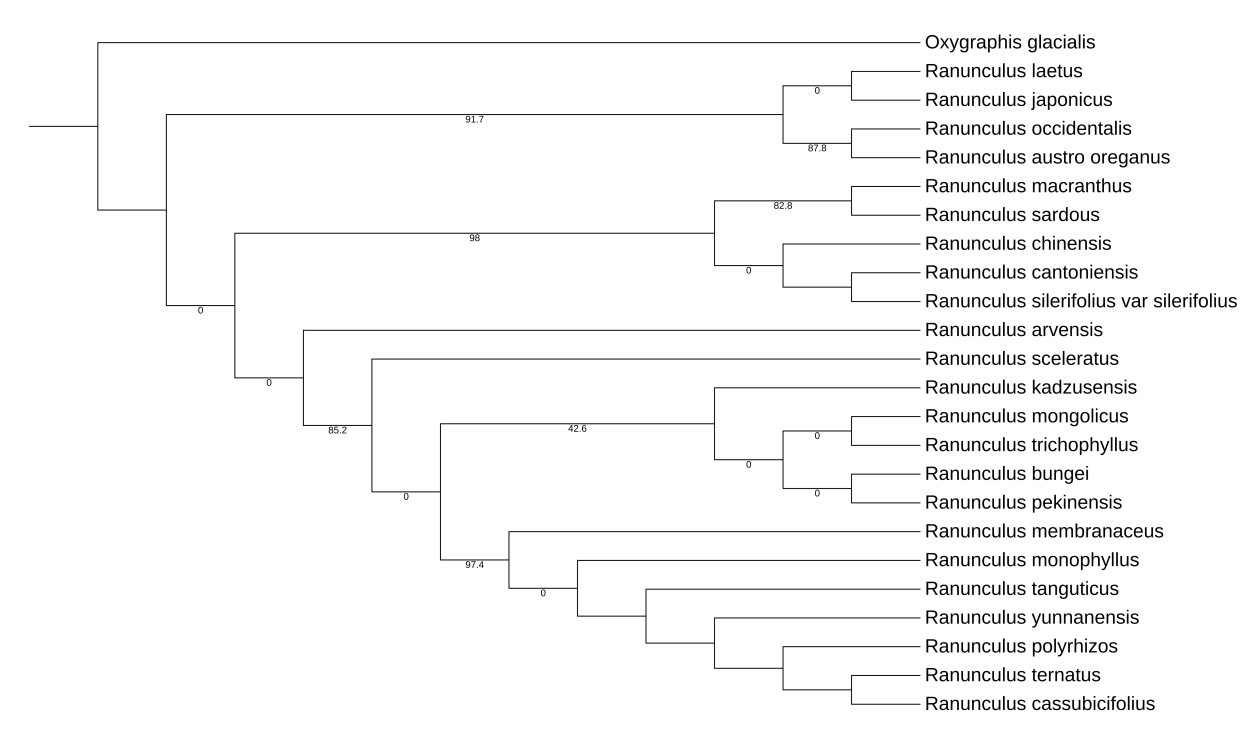


Figure S16. Phylogenetic tree of 23 *Ranunculus* species based on the *ndhA-ndhI* IGS regions.


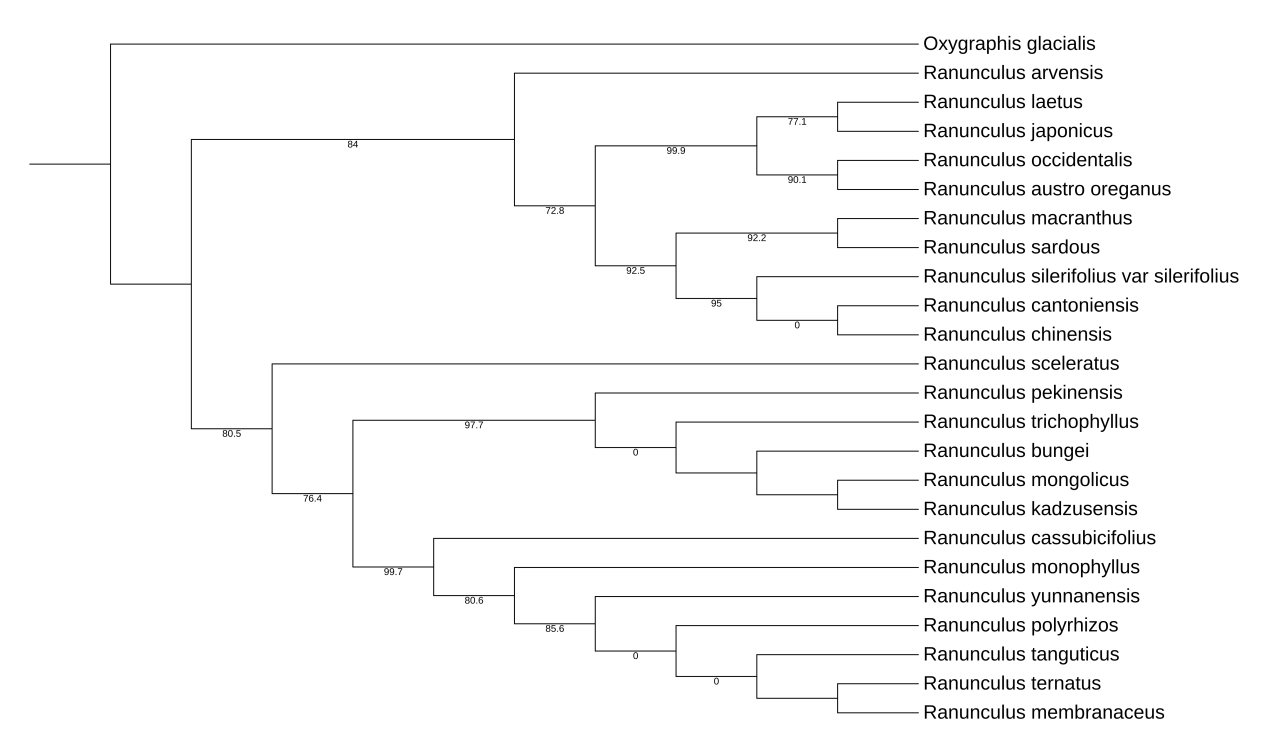


Figure S17. Phylogenetic tree of 23 *Ranunculus* species based on the *ndhE-ndhG*

IGS regions.


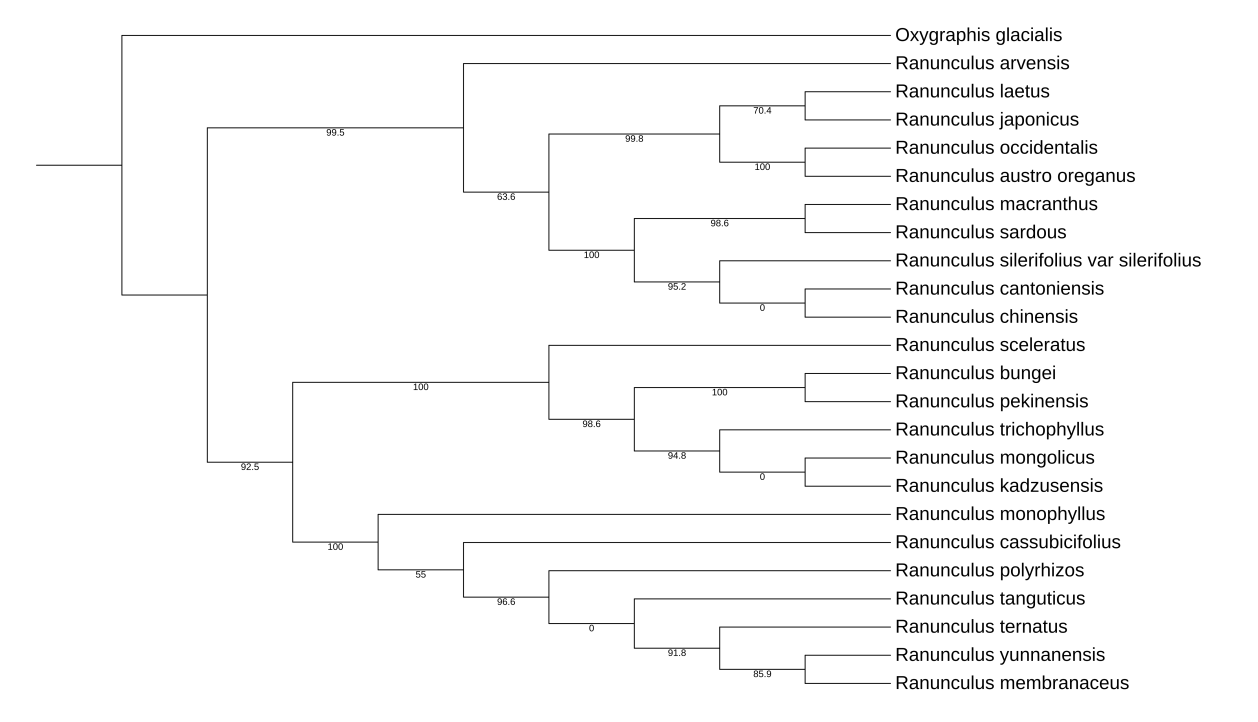


Figure S18. Phylogenetic tree of 23 *Ranunculus* species based on the *ndhF-rpl32*

IGS regions.


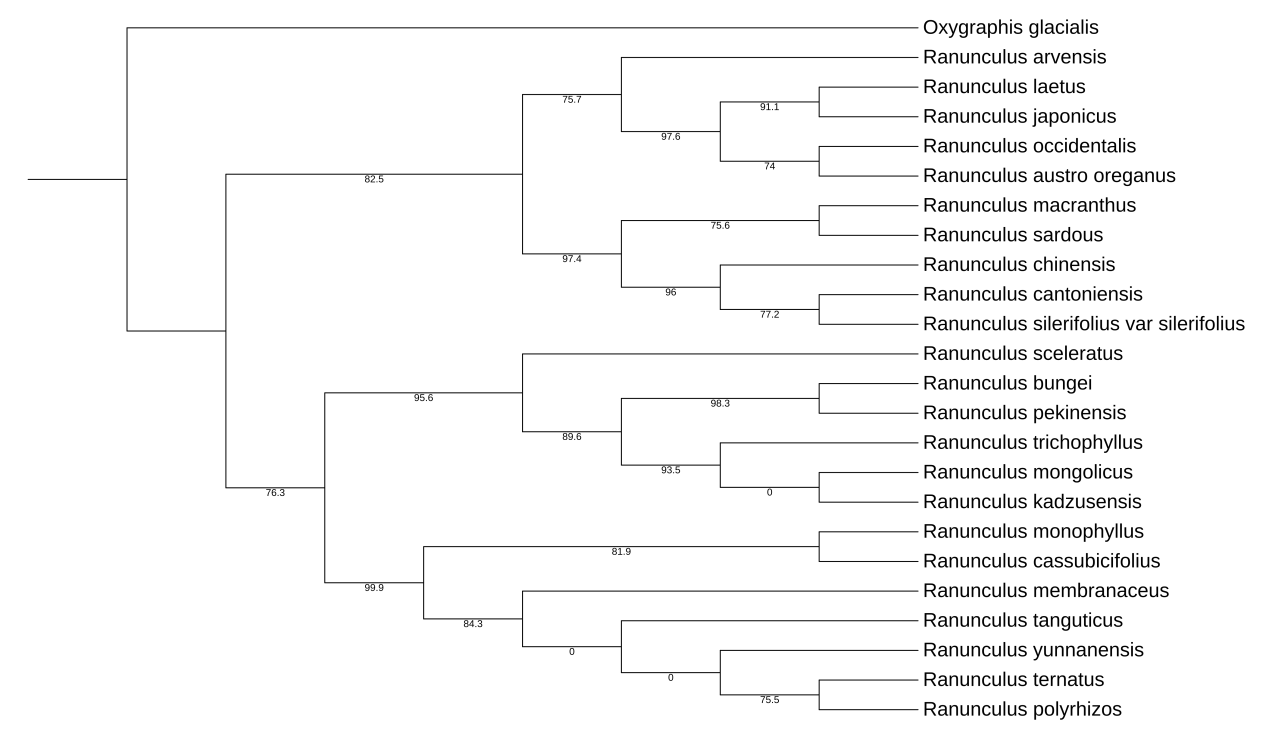


Figure S19. Phylogenetic tree of 23 *Ranunculus* species based on the *ndhG-ndhI*

IGS regions.


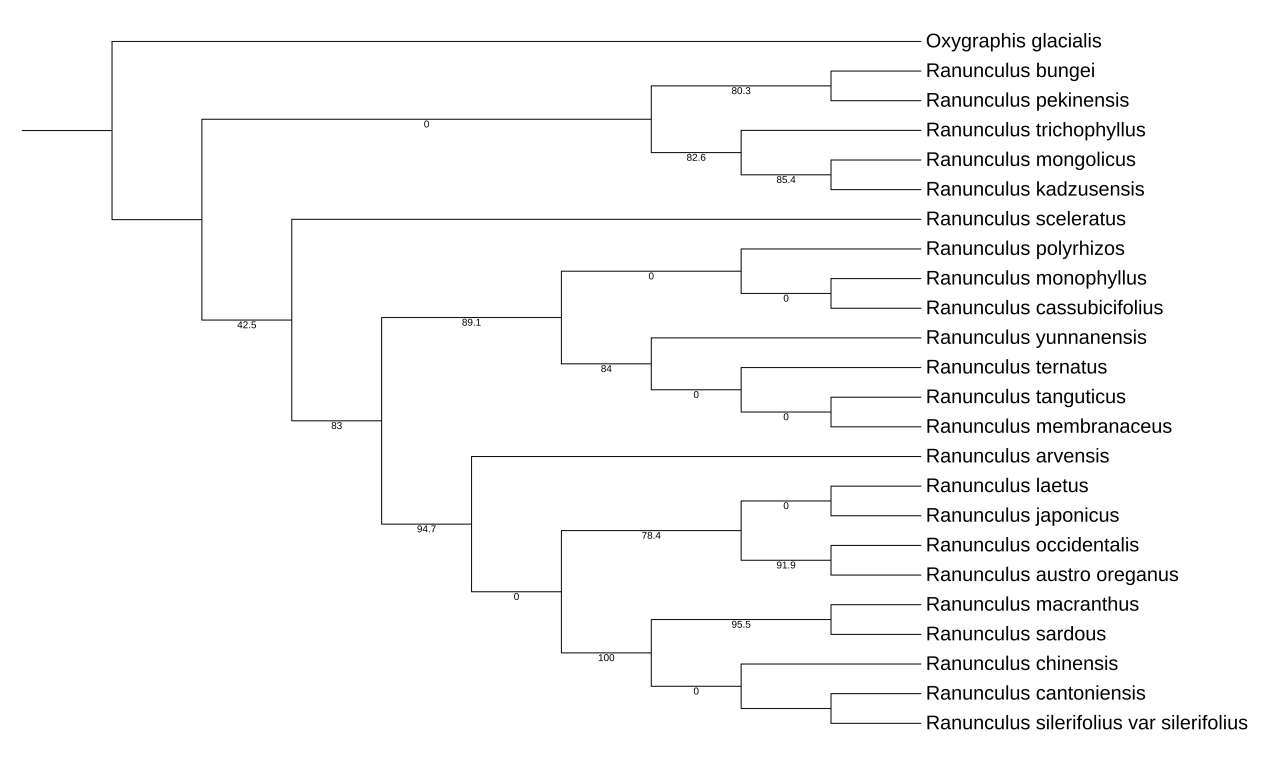


Figure S20. Phylogenetic tree of 23 *Ranunculus* species based on the *petG-trnW*

IGS regions.


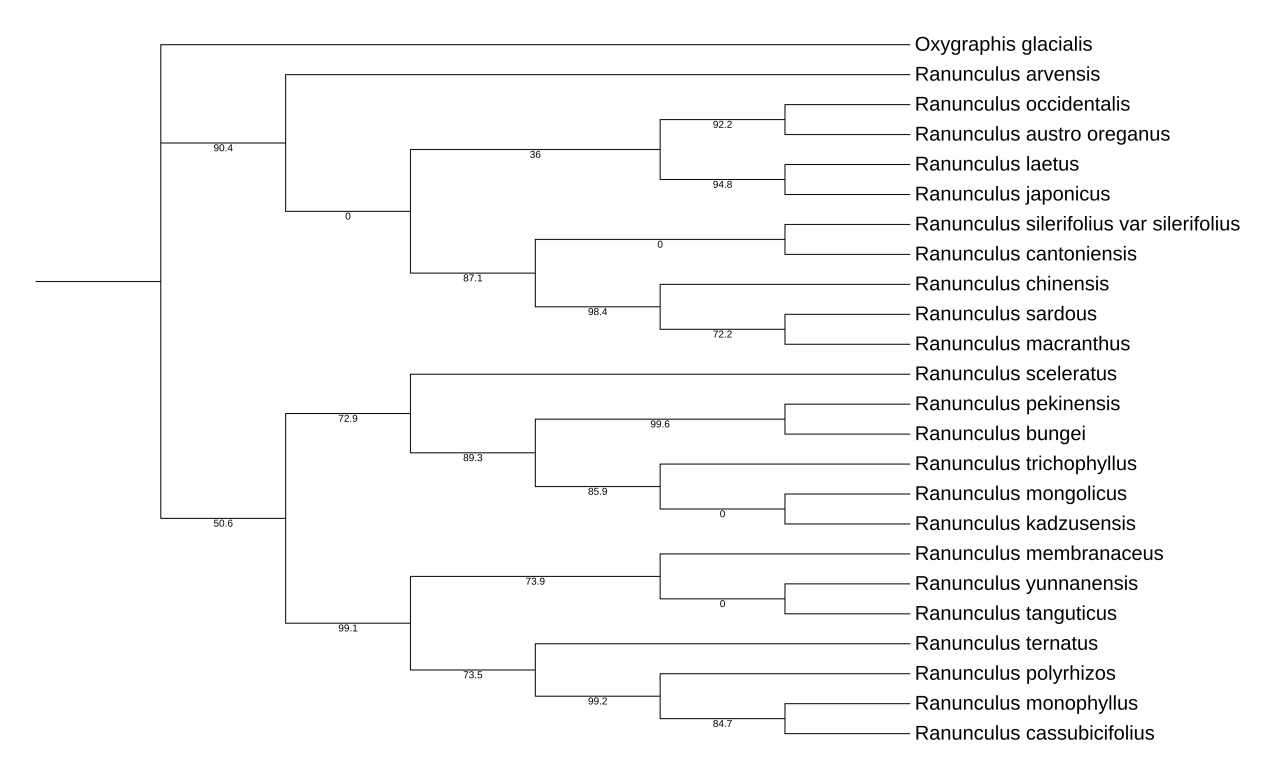


Figure S21. Phylogenetic tree of 23 *Ranunculus* species based on the *psbA-trnH*

IGS regions.


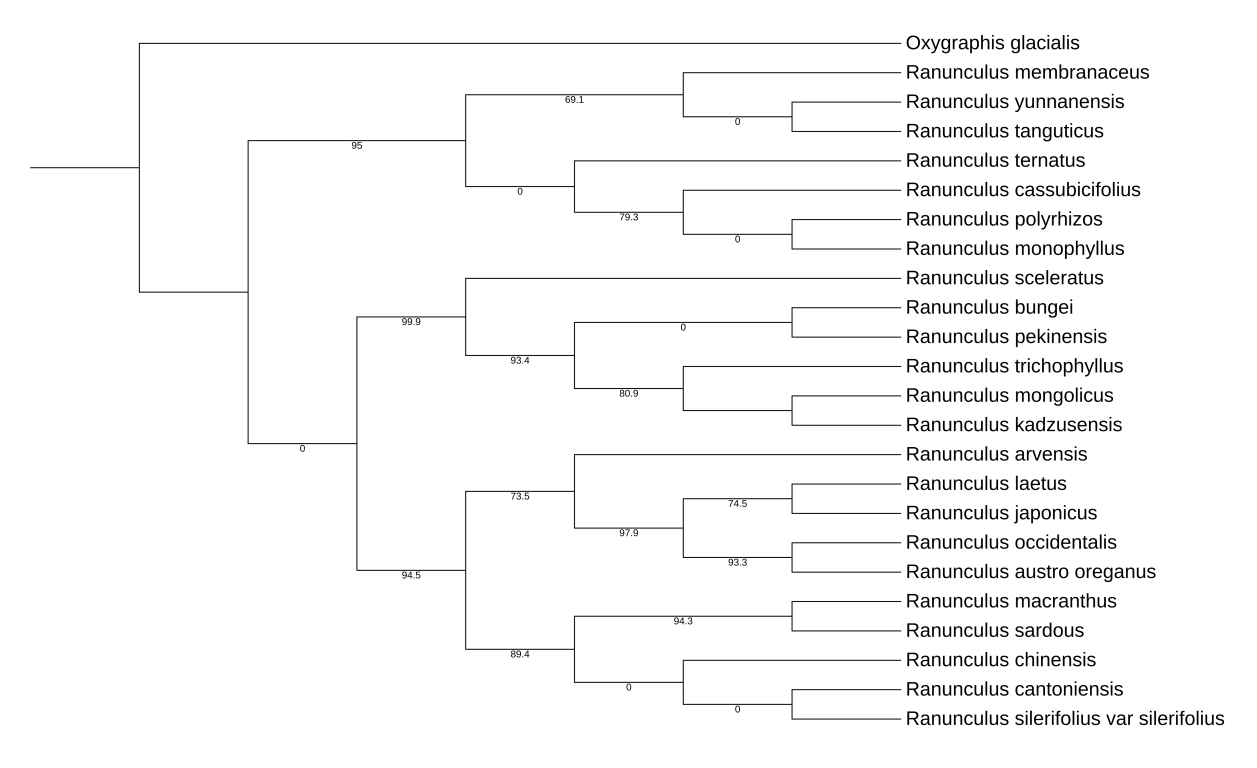


Figure S22. Phylogenetic tree of 23 *Ranunculus* species based on the *rpl14-rps8*

IGS regions.


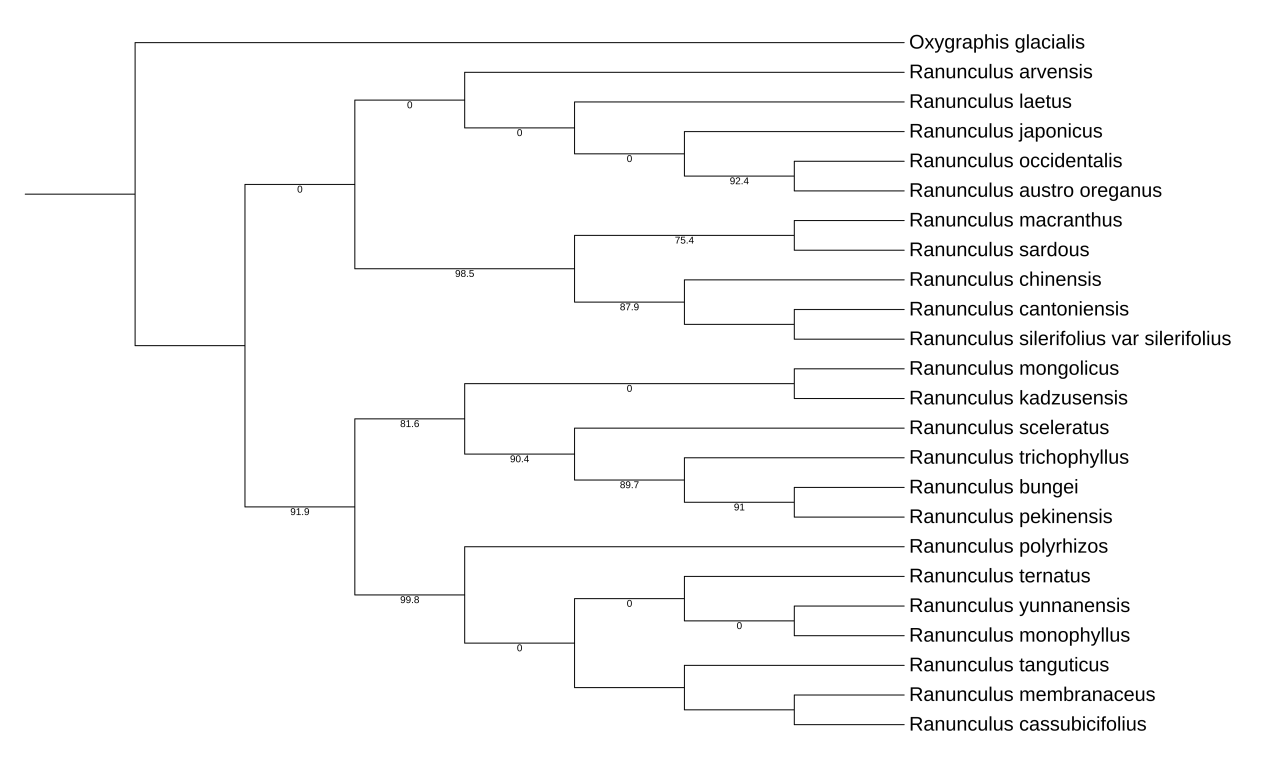


Figure S23. Phylogenetic tree of 23 *Ranunculus* species based on the *rpl16-rps3*

IGS regions.


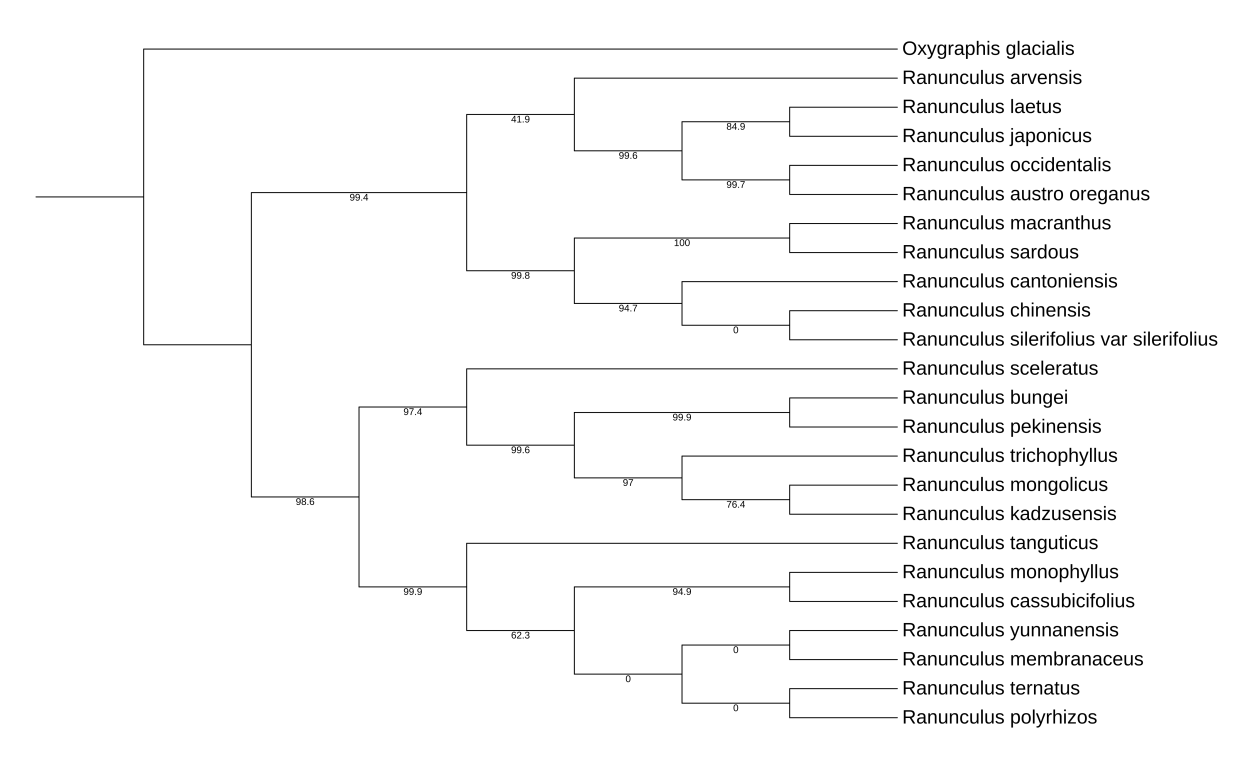


Figure S24. Phylogenetic tree of 23 *Ranunculus* species based on the *rpl32-trnL*

IGS regions.
